# Penalized likelihood estimation of a mixture cure Cox model with partly interval censoring—An application to thin melanoma

**DOI:** 10.1002/sim.9415

**Published:** 2022-04-26

**Authors:** Annabel Webb, Jun Ma, Serigne N. Lô

**Affiliations:** ^1^ Department of Mathematics and Statistics Macquarie University Sydney New South Wales Australia; ^2^ Melanoma Institute Australia The University of Sydney North Sydney New South Wales Australia; ^3^ Faculty of Medicine and Health The University of Sydney Sydney New South Wales Australia; ^4^ Institute for Research and Medical Consultations (IRMC) Imam Abdulrahman Bin Faisal University Dammam Saudi Arabia

**Keywords:** asymptotic variance, constrained optimization, direct likelihood maximization, mixture cure model, penalized likelihood

## Abstract

Time‐to‐event data in medical studies may involve some patients who are cured and will never experience the event of interest. In practice, those cured patients are right censored. However, when data contain a cured fraction, standard survival methods such as Cox proportional hazards models can produce biased results and therefore misleading interpretations. In addition, for some outcomes, the exact time of an event is not known; instead an interval of time in which the event occurred is recorded. This article proposes a new computational approach that can deal with both the cured fraction issues and the interval censoring challenge. To do so, we extend the traditional mixture cure Cox model to accommodate data with partly interval censoring for the observed event times. The traditional method for estimation of the model parameters is based on the expectation‐maximization (EM) algorithm, where the log‐likelihood is maximized through an indirect complete data log‐likelihood function. We propose in this article an alternative algorithm that directly optimizes the log‐likelihood function. Extensive Monte Carlo simulations are conducted to demonstrate the performance of the new method over the EM algorithm. The main advantage of the new algorithm is the generation of asymptotic variance matrices for all the estimated parameters. The new method is applied to a thin melanoma dataset to predict melanoma recurrence. Various inferences, including survival and hazard function plots with point‐wise confidence intervals, are presented. An R package is now available at Github and will be uploaded to R CRAN.

## 1 INTRODUCTION

The prognosis of patients diagnosed with thin primary cutaneous melanomas, defined as a Breslow tumor thickness ≤1.0 mm, is generally excellent. The 10‐year melanoma‐specific survival rates range between 82% and 98%. Based on the presence of a large number of long‐term survivors in published Kaplan‐Meier survival curves, one can reasonably consider that some individuals will never experience cancer recurrence and therefore are cured.^1^, ^2^ Being able to identify cured and non‐cured patients is of high importance to thin melanoma patients for their management decisions and appropriate follow‐up schedules.

Multiple studies have analyzed death due to melanoma to evaluate the prognosis or predictive value of patient and tumor characteristics at the time of diagnosis. However, very few studies have analyzed time to melanoma recurrence in this context. To our view, one reason for why time to recurrence is underused is because the exact time of recurrence is challenging to determine (interval‐censored). To alleviate this issue, the time at which recurrence is diagnosed is usually analyzed instead, but the interpretation is still made in terms of the time to recurrence. Alternative methods that model the recurrence time need to be developed and made accessible to the community for more accurate prognospis and predictive analyses.

In standard survival analysis, patients who do not experience the event of interest (eg, death or recurrence) during the follow‐up time are simply right censored. However, in some circumstances, some individuals might be cured and therefore would never experience the event no matter the length of follow‐up. Those patients constitute a cured fraction and should be considered as such. In general, a cured fraction is never observed directly; its information is contained in the right‐censored times of a dataset. A common feature for a dataset having a cured fraction is the presence of a group of long‐term survivors. Ignoring the cured fraction, and thus treating all the observed right censored times as true right censoring of event times, may lead to biased inferences.

The most popular approach to deal with a cured fraction is called the mixture cure model, and was first proposed by Farewell.^3^ This model considers the population of interest to be a mixture of two sub‐populations, one of which is susceptible to the event of interest, and another which is cured. Models for these two components are called latency and incidence models, respectively. The incidence is most commonly modeled using a logistic regression as in Reference 3. For the latency there has been consideration of parametric survival models, as well as much focus on the use of semi‐parametric survival models such as the Cox model.^4^, ^5^, ^6^, ^7^, ^8^ Such a model is appropriate in cases where there is evidence of a possible cured fraction in the data, based on biological feasibility and the presence of a large number of long‐term survivors in the sample even after a lengthy follow‐up time (see discussion in, for instance, Reference 9). When fitted to a sample without a cured fraction, the mixture cure model reduces to a standard Cox proportional hazards model.

In this article, we propose to extend the mixture cure Cox model to accommodate data with a more general partly interval censoring scheme that consists of exact event times and left, interval and right censoring times. We will develop a maximum penalized likelihood (MPL) method to estimate the model parameters, including the logistic regression parameters, the Cox regression coefficients and nonparametric baseline hazard. A penalty function is used for a smooth estimate of the baseline hazard.

Parameter estimation for a mixture cure Cox model is difficult to carry out using Cox's partial likelihood. A variety of mixture cure Cox model estimation methods for event times with right censoring have been proposed, including References 10, 11, 12. In more recent work, there has also been consideration of interval censoring.^13^, ^14^, ^15^, ^16^, ^17^, ^18^ Previous research on this topic has only rarely considered a smooth estimate of the baseline hazard function. The method in Reference 18 allows for a piecewise linear approximation to the cumulative baseline hazard of a mixture cure model for interval‐censored data. Alternatively, Corbière et al^19^ consider a smooth estimate of the baseline hazard function directly via the use of a penalized likelihood, but was limited to right censored samples only.

This article sits within a wider body of research concerning penalized likelihood estimation of proportional hazards models.^20^, ^21^, ^22^ Generally, this approach introduces a roughness penalty term to the likelihood to produce a smooth baseline hazard function estimate that is approximated using a set of non‐negative basis functions. Ma et al^23^ demonstrate that a MPL approach can easily incorporate partly interval censored data. To date, there has been extremely limited consideration of methods for fitting a proportional hazards mixture cure model to partly interval censored data that provides a smooth estimate of the baseline hazard function. This article aims to address this gap by drawing on the MPL method for fitting a proportional hazards model to partly interval censored data.

The rest of this article is organized as follows. In Section 2, the mixture cure Cox model is introduced. It also explains an approximation of the baseline hazard function using M‐spline basis functions, and presents the penalized likelihood function. In Section 3, we lay out the simultaneous estimation of the regression parameters and baseline hazard function using an alternating algorithm for the constrained MPL estimation. An automatic smoothing parameter estimation procedure is also presented in this section. Section 4 details the asymptotic properties of the estimates. These asymptotic results enable inferences on both regression parameters and survival quantities without computationally intensive methods such as bootstrapping. In Section 5, we report and discuss the results of two simulation studies, and in Section 6, an application to the thin melanoma study is illustrated. Finally, concluding remarks are included in Section 7.

## 2 MIXTURE CURE COX MODELS AND PENALIZED LIKELIHOOD

Let $Y_{i}$ be a random variable denoting the time to the event of interest of individual i (i=1,…,n), where it is possible that some individuals are not susceptible to experiencing the event. Under a scenario of partly interval censored survival data, we may observe event times and also right, left and interval censoring times. In this article, the recorded survival time for each individual i will be denoted as a random vector $T_{i}={\left(T_{i}^{L},T_{i}^{R}\right)}^{T}$ containing $Y_{i}$, where $T_{i}^{R}\geq T_{i}^{L}\geq 0$, and we may have $T_{i}^{R}=+\infty$ for a right‐censored time and $T_{i}^{L}=0$ for a left‐censored time. If the event time $Y_{i}$ is observed directly, then we have $T_{i}^{L}=T_{i}^{R}$. Let $U_{i}$ be an unobserved random variable where $U_{i}=1$ indicates that individual i is susceptible to the event or is non‐cured (ie, patient will experience the event of interest given a sufficient follow‐up), and $U_{i}=0$ indicates otherwise (ie, patient is cured after treatment and will never experience the event of interest regardless of the length of his/her follow‐up). Using a Cox proportional hazards regression model, we can denote the hazard function of $Y_{i}$ in the non‐cured fraction as

(1) $$h(t|U_{i}=1,x_{i})=h_{0}(t)\exp\{x_{i}^{T}\gamma \},$$

where $h_{0}(t)$ is an unknown baseline hazard function, $x_{i}$ is a vector for the values of of covariates, and γ is a q‐vector of proportional hazards regression parameters. We may denote $h(t|U_{i}=1,x_{i})$ simply as $h(t|x_{i})$ when there is no confusion. Additionally, we can model the probability of having $U_{i}=1$ using a logistic regression model, such that

(2) $$\pi (z_{i})=\frac{\exp\{z_{i}^{T}\beta \}}{1+\exp\{z_{i}^{T}\beta \}},$$

where $\pi (z_{i})=P(U_{i}=1|z_{i})$ is the probability of being non‐cured, $z_{i}$ is a set of covariates that may be identical to, or be completely different from $x_{i}$. It is also possible that $z_{i}$ share some components with $x_{i}$. In (2), β is a p‐vector for the logistic regression parameters.

Now, we can specify the survival function for the whole population, consisting of both the cured and non‐cured fractions, as

(3) $$S_{pop}(t|x_{i},z_{i})=\pi (z_{i})S(t|U_{i}=1,x_{i})+(1-\pi (z_{i})).$$

Clearly, when $\pi (z_{i})=1$ (ie, all members of the population are susceptible to the event and there is no cured fraction) the above survival function reduces to that of a standard Cox model. Also, we may denote the conditional survival function $S(t|U_{i}=1,x_{i})$ simply as $S(t|x_{i})$ when its meaning is clear in the context.

For our derivations, it is convenient to denote the information of event, left, right, and interval censoring times using a set of indicator values for each i. Let $\delta_{i},\delta_{i}^{L},\delta_{i}^{R}$, and $\delta_{i}^{I}$ be, respectively, the indicators for event time, left, right, and interval censoring time for i. Thus, for each i, the set of observed information available is $\left(t_{i}^{L},t_{i}^{R},x_{i}^{T},z_{i}^{T},\delta_{i},\delta_{i}^{R},\delta_{i}^{L},\delta_{i}^{I}\right)$. Note that for the survival times corresponding to event, left or interval censoring we have $U_{i}=1$. Conversely, for the right‐censored times their $U_{i}$ values are unknown.

Similar to the method of sieves (eg, Reference 24), the nonparametric baseline hazard function $h_{0}(t)$ can be approximated using some m non‐negative basis functions, where m is a positive integer, such that

(4) $$h_{0}(t)=\overset{m}{\underset{u=1}{\sum }}\theta_{u}\psi_{u}(t),$$

where each coefficient $\theta_{u}\geq 0$ and each $\psi_{u}(t)$ is a non‐negative basis function. Let the vector of $\theta_{u}$ be denoted by θ. Here, the baseline hazard function will be approximated using cubic M‐splines, following from previous work on MPL estimation of a proportional hazards model such as References 20 and 23.

One convenience of using M‐splines to approximate $h_{0}(t)$ is that ensuring the estimate of the baseline hazard function is non‐negative requires only that the spline coefficient vector θ be non‐negative. Another benefit is that it makes the computation of the cumulative baseline hazard function $H_{0}(t)$ straightforward: $H_{0}(t)=\sum_{u=1}^{m}\theta_{u}\Psi_{u}(t)$, where $\Psi_{u}(t)$ are I‐splines.^25^ M‐splines and their corresponding I‐splines are readily available in, for example, the R splines2 package.

The proposed method in this article is to estimate the three parameter vectors, β, γ, and θ simultaneously by maximizing a penalized likelihood function. Let η=(β,γ,θ).

Under independent interval censoring (eg, Reference 26), the log‐likelihood is given by

(5) $$\begin{array}{ll} l(\eta ) & =\overset{n}{\underset{i=1}{\sum }}\{\delta_{i}[\ln\pi (z_{i})+\ln h_{0}(t)+x_{i}^{T}\gamma +\ln S(t_{i}|x)]+\delta_{i}^{R}\ln(1-\pi (z_{i})+\pi (z_{i})S(t_{i}|x_{i}))\\ & \;+\delta_{i}^{L}[\ln\pi (z_{i})+\ln(1-S(t_{i}|x_{i}))]+\delta_{i}^{I}[\ln\pi (z_{i})+\ln(S(t_{i}^{L}|x_{i})-S(t_{i}^{R}|x_{i}))]\}. \end{array}$$

Direct maximization of l(η) for estimation of θ is not ideal. This is because: (1) $h_{0}(t)$ is usually a smooth function and this information should be incorporated into the estimation of θ; and (2) it is possible that knots selected to approximate $h_{0}(t)$ may not be optimal, where particularly some knots may be unnecessary so that their corresponding $\theta_{u}$'s should be zero. We use a penalized log‐likelihood to obtain a smooth estimate for $h_{0}(t)$ and to force the unnecessary $\theta_{u}$'s close to zero. We adopt a roughness quadratic penalty function and the penalized likelihood is now given by

(6) Φ(η)=l(η)−λJ(η),

where J(η) is a roughness penalty function and λ≥0 is a smoothing parameter. The roughness penalty is given by $\int h_{0}^{\prime \prime }{\left(v\right)}^{2}dv$. Given $h_{0}(t)$ is now given by (4), we can conveniently express this roughness penalty as $J(\eta )=\theta^{T}R\theta$, where R is an m×m matrix with the (u,v)th element given by $r_{u,v}=\int \psi_{u}^{\prime \prime }(t)\psi_{v}^{\prime \prime }(t)dt$.

We comment that in general the above roughness penalty is effective for imposing smoothness on $h_{0}(t)$ but less ideal for constraining unnecessary $\theta_{u}$ to zero. A composite penalty employing both quadratic and $l_{1}$‐norm (equivalent to lasso) penalty could be more efficient. Further investigations are necessary to explore this option.

## 3 PENALIZED LIKELIHOOD ESTIMATION

### 3.1 A constrained optimization algorithm

The MPL estimate of η, denoted by $\hat{\eta }$, is obtained by

(7) $$\hat{\eta }=\underset{\theta \geq 0}{\max}\;\Phi (\eta ).$$

Given the constraint that θ≥0, we have the following Karush‐Kuhn‐Tucker (KKT) conditions for a constrained optimal solution:

$$\begin{array}{ll} & \frac{\partial \Phi (\eta )}{\partial \beta_{t}}=0,\;\;\frac{\partial \Phi (\eta )}{\partial \gamma_{j}}=0;\\ & \frac{\partial \Phi (\eta )}{\partial \theta_{u}}=0\;\text{if}\;\theta_{u}>0,\;\;\frac{\partial \Phi (\eta )}{\partial \theta_{u}}<0\;\text{if}\;\theta_{u}=0. \end{array}$$

These conditions are solved iteratively using an algorithm similar to the Newton‐MI algorithm of Reference 23. This algorithm requires the score vector and the Hessian matrix for β and γ, but for θ it only demands its score vector. Details of score vectors and Hessian matrices can be found in the Supplementary Materials.

Before describing this algorithm we first introduce some notations. Let $\beta^{\left(k\right)}$, $\gamma^{\left(k\right)}$, and $\theta^{\left(k\right)}$ be, respectively, the estimates of β, γ, and θ at iteration k. Also, for any function a(x), we let $a{\left(x\right)}^{+}$ and $a{\left(x\right)}^{-}$ be respective the positive and negative components of a(x), so that $a{\left(x\right)}^{+}-a{\left(x\right)}^{-}=a(x)$. Iteration k+1 of our algorithm is obtained in a three‐step process as follows. First, obtain $\beta^{\left(k+1\right)}$ using a modified Newton algorithm:

(8) $$\beta^{\left(k+1\right)}=\beta^{\left(k\right)}+\omega_{1}^{\left(k\right)}{\left[-\frac{\partial^{2}\Phi (\beta^{\left(k\right)},\gamma^{\left(k\right)},\theta^{\left(k\right)})}{\partial \beta \partial \beta^{T}}\right]}^{-1}\left[\frac{\partial \Phi (\beta^{\left(k\right)},\gamma^{\left(k\right)},\theta^{\left(k\right)})}{\partial \beta }\right],$$

where $\omega_{1}\in (0,1]$ is the line search step size used to ensure that $\Phi (\beta^{\left(k+1\right)},\gamma^{\left(k\right)},\theta^{\left(k\right)})\geq \Phi (\beta^{\left(k\right)},\gamma^{\left(k\right)},\theta^{\left(k\right)})$. The value of the line search step size can be determined by using, for instance, Armijo's rule.^27^ Second, we compute $\gamma^{\left(k+1\right)}$ using again a modified Newton algorithm:

(9) $$\gamma^{\left(k+1\right)}=\gamma^{\left(k\right)}+\omega_{2}^{\left(k\right)}{\left[-\frac{\partial^{2}\Phi (\beta^{\left(k+1\right)},\gamma^{\left(k\right)},\theta^{\left(k\right)})}{\partial \gamma \partial \gamma^{T}}\right]}^{-1}\left[\frac{\partial \Phi (\beta^{\left(k+1\right)},\gamma^{\left(k\right)},\theta^{\left(k\right)})}{\partial \gamma }\right],$$

where $\omega_{2}$ is defined similarly to $\omega_{1}$. Finally, we get the update $\theta^{\left(k+1\right)}$ using the multiplicative‐iterative algorithm:

(10) $$\theta^{\left(k+1\right)}=\theta^{\left(k\right)}+\omega_{3}^{\left(k\right)}D^{\left(k\right)}\frac{\partial \Phi (\beta^{\left(k+1\right)},\gamma^{\left(k+1\right)},\theta^{\left(k\right)})}{\partial \theta },$$

where $\omega_{3}$ is defined similarly to $\omega_{1}$ and $\omega_{2}$ and $D^{\left(k\right)}$ is a diagonal m×m matrix with elements $\theta_{u}^{\left(k\right)}/d_{u}^{\left(k\right)}$ for u=1,…,m, and where

$$d_{u}={\left[\frac{\partial l(\beta ,\gamma ,\theta )}{\partial \theta_{u}}\right]}^{-}+\lambda {\left[\frac{\partial J(\theta )}{\partial \theta_{u}}\right]}^{+}+\xi .$$

Referring to the Supplementary Materials for the score vector of θ, we can see that for the problem considered in this article, $d_{u}$ becomes

$$\begin{array}{ll} d_{u} & =\delta_{i}\Psi_{u}(t_{i})e^{x_{i}^{T}\gamma }+\delta_{i}^{R}\frac{\pi (z_{i})S(t_{i}|x_{i})\Psi_{u}(t_{i})}{1-\pi (z_{i})+\pi (z_{i})S(t_{i}|x_{i})}e^{x_{i}^{T}\gamma }\\ & \;+\delta_{i}^{I}\frac{S(t_{i}^{L}|x_{i})\Psi_{u}(t_{i}^{L})}{S(t_{i}^{L}|x_{i})-S(t_{i}^{R}|x_{i})}e^{x_{i}^{T}\gamma }+\lambda {\left[\frac{\partial J(\theta )}{\partial \theta_{u}}\right]}^{+}+\xi_{u}. \end{array}$$

Note that $\xi_{u}\geq 0$ is a small constant included simply to avoid the numerical issue of a zero denominator in the calculation of $D^{\left(k\right)}$ and this value does not have any impact on the final solution for θ.

### 3.2 Estimation of the smoothing parameter

A marginal likelihood method for the automatic selection of the smoothing parameter, previously outlined in, for example, References 23 and 28, can be implemented to the model of this article. In this method, the penalty function $J(\eta )=\lambda \theta^{T}R\theta$ is related to a normal prior distribution for the vector θ with $\theta ∼N(0_{m\times 1},\sigma_{\theta }^{2}R^{-1})$, where $\sigma_{\theta }^{2}=1/2\lambda$. We can then obtain the log‐posterior:

(11) $$l_{p}(\beta ,\gamma ,\theta )=-\frac{m}{2}\log\sigma_{\theta }^{2}+l(\beta ,\gamma ,\theta )-\frac{1}{2\sigma_{\theta }^{2}}\theta^{T}R\theta .$$

The marginal likelihood for $\sigma_{\theta }^{2}$ may be difficult to obtain directly, and as such we can approximate it using Laplace's method. Applying the Laplace approximation and substituting in the MPL estimates of β, γ, and θ, we can obtain the approximated log‐marginal likelihood for $\sigma_{\theta }^{2}$:

(12) $$l_{m}(\sigma_{\theta }^{2})\approx -\frac{m}{2}\log\sigma_{\theta }^{2}+l(\hat{\beta },\hat{\gamma },\hat{\theta })-\frac{1}{2\sigma_{\theta }^{2}}{\hat{\theta }}^{T}R\hat{\theta }-\frac{1}{2}\log|\hat{G}+Q(\sigma_{\theta }^{2})|,$$

where $\hat{G}$ is the negative Hessian matrix from l(β,γ,θ) evaluated at the MPL estimates $\hat{\beta }$, $\hat{\gamma }$, and $\hat{\theta }$, and

$$Q(\sigma_{\theta }^{2})=\left[\begin{array}{cc} 0 & 0\\ 0 & \frac{1}{\sigma_{\theta }^{2}}R \end{array}\right].$$

An approximate maximum marginal likelihood solution for $\sigma_{\theta }^{2}$ is:

(13) $${\hat{\sigma }}_{\theta }^{2}=\frac{{\hat{\theta }}^{T}R\hat{\theta }}{m-\nu },$$

where $\nu =\text{tr}\{{\left(\hat{G}+Q({\hat{\sigma }}_{\theta }^{2})\right)}^{-1}Q({\hat{\sigma }}_{\theta }^{2})\}$, which can be considered as equivalent to the model degrees of freedom. Given that the estimates of β, γ, and θ depend on $\sigma_{\theta }^{2}$, this approximate solution for $\sigma_{\theta }^{2}$ allows for the development of an iterative procedure with two steps. First, with a current $\sigma_{\theta }^{2}$, the corresponding MPL estimates for β, γ, and θ are obtained. Then, $\sigma_{\theta }^{2}$ is updated using the current ${\hat{\sigma }}_{\theta }^{2}$, and the just obtained $\hat{\beta }$, $\hat{\gamma }$, and $\hat{\theta }$ on the right‐hand side of (13). These two steps are repeated until ν is stabilized, such as the difference between two consecutive ν values is less than 1.

## 4 ASYMPTOTIC PROPERTIES AND INFERENCE

Development of the asymptotic properties of the proposed model allows for large sample inference to be conducted without reliance on bootstrapping or other computationally intensive methods. Following from Reference 23, it is possible to demonstrate asymptotic consistency for the MPL estimates of both sets of regression parameters, β and γ, and the baseline hazard function $h_{0}(t)$. We adopt $\beta_{0}$, $\gamma_{0}$, and $h_{00}(t)$ to denote the true model parameters (ie, the parameters that gave rise to the observed data). Theorem 1 states this asymptotic property; the proofs can be found in the Supplementary Materials.

Theorem 1 *Assume that conditions B1 to B4 (see the Supplementary Materials) hold. Assume that* $h_{0}(t)$ *is bounded and has some number* r≥1 *derivatives over the interval* [a,b]. *Assume that* $m=n^{v}$ *, where* 0<v<1. *Then, when* n→∞ *,*

*1*. $||\hat{\beta }-\beta_{0}||\to 0$ *almost surely,*
*2*. $||\hat{\gamma }-\gamma_{0}||\to 0$ *almost surely, and*
*3*. $\sup_{t\in [a,b]}|{ĥ}_{0}(t)-h_{00}(t)|\to 0$ *almost surely*.

Additionally, it is desirable to develop asymptotic normality results for all three parameters, β, γ, and θ as this allows for inference to be made not only on regression parameters but also on other quantities, such as survival probabilities. In order to develop these results, however, it is necessary to restrict m to be a finite number, similar to References 23 and 29. Note that this fixed m is not predetermined as it depends on the given sample size n. Usually, a practical guide for m is $m=n_{0}^{1/3}$. where $n_{0}$ denotes the non‐right censored samples size.

Another issue we face when developing asymptotic normality results is that we must take into account the possibility of encountering active constraints in the estimation of θ≥0. This is particularly likely to occur when the number of knots is larger than strictly necessary, or some knots are placed at non‐important locations. The penalty function will push the corresponding $\theta_{u}$ to zero.^23^ Ignoring active constraints often leads to undesirable results, such as negative variances.

Recall that we have defined the parameter vector η=(β,γ,θ), which has a length of p+q+m, and that we can express the penalized likelihood function in terms of η such that

Φ(η)=l(η)−λJ(η).

Let $\hat{\eta }$ be the MPL estimate of η. Let the true value of η be represented by $\eta_{0}$. Without loss of generality, we assume that the estimates of first r elements of θ are zero, and so that they are actively constrained. Define

(14) $$U={\left[0_{(m-r+p+q)\times r},I_{\left(m-r+p+q)\times (m-r+p+q\right)}\right]}^{T},$$

where 0 is a matrix of zeros, I is an identity matrix. Clearly, $U^{T}U=I_{\left(m-r+p+q)\times (m-r+p+q\right)}$ is satisfied. Theorem 2 states the asymptotic normality results, with relevant proofs in the Supplementary Materials.

Theorem 2 *Let* $\mu_{n}=\lambda /n$. *Assume that* $\mu_{n}=o(n^{1/2})$ *and that we have the first* r *active constraints in the MPL estimate of* θ. *Define matrix* U *as above. Let*

$$F(\eta )=-E_{\eta_{0}}\left[\underset{n\to \infty }{\lim}n^{-1}\frac{\partial^{2}l(\eta )}{\partial \eta \partial \eta^{T}}\right],$$

*where matrix* U *is defined in (* *14* *). Under these conditions, when* n→∞ *,* $\sqrt{n}(\hat{\eta }-\eta_{0})$ *converges in distribution to* $𝒩(0,\tilde{F}{\left(\eta_{0}\right)}^{-1})$ *, where* $\tilde{F}{\left(\eta_{0}\right)}^{-1}=U{\left(U^{T}F(\eta )U\right)}^{-1}U^{T}$.

In order to implement the result of Theorem 2, it is necessary to define a method for identifying active constraints when they arise in the MPL estimation of θ. The method used here follows that proposed by Ma et al.^23^ Active constraints can be identified by inspecting both the value of ${\hat{\theta }}_{u}$ and the corresponding gradient for each u. After the Newton‐MI algorithm has reached convergence, some ${\hat{\theta }}_{u}$ may be exactly zero with negative gradients, and thus are clearly subject to an active constraint. Furthermore, there may be some ${\hat{\theta }}_{u}$ that are very close to, but not exactly, zero. For these ${\hat{\theta }}_{u}$, a corresponding negative gradient value is indicative that they are also subject to an active constraint. In practice, active constraints are defined where, for a given u, ${\hat{\theta }}_{u}<10^{-3}$ and the corresponding gradient is less than −ε, where ε is a positive threshold value such as $10^{-2}$. After the indices associated with active constraints are identified, obtaining the matrix $\tilde{F}{\left(\eta_{0}\right)}^{-1}$ is a very straightforward computation. The matrix $U^{T}F(\eta )U$ is obtained by removing the rows and columns of F(η) associated with the active constraints. The result is then inverted, and then padded with zeros in the deleted rows and columns to obtain $\tilde{F}{\left(\eta_{0}\right)}^{-1}$.

To make use of these asymptotic results for inference on finite samples, it is necessary to approximate the distribution for $\hat{\eta }$ when n is large. Doing so also incorporates nonzero values for the smoothing parameter λ into the inference on the parameter estimates. The necessary results are presented below in Corollary 1.

Corollary 1 *Assume that the smoothing parameter* λ≪n. *Define*

$$A{\left(\hat{\eta }\right)}^{-1}=U{\left(U^{T}\left(\frac{\partial^{2}l(\hat{\eta })}{\partial \eta \partial \eta^{T}}+\lambda \frac{\partial^{2}J(\hat{\eta })}{\partial \eta \partial \eta^{T}}\right)U\right)}^{-1}U^{T}.$$

*Then, when* n *is large, the distribution for the MPL estimate* $\hat{\eta }-\eta_{0}$ *can be approximated by a multivariate normal distribution having mean zero and covariance matrix*

$$\hat{\mathrm{var}}(\hat{\eta })=A{\left(\hat{\eta }\right)}^{-1}\frac{\partial^{2}l(\hat{\eta })}{\partial \eta \partial \eta^{T}}A{\left(\hat{\eta }\right)}^{-1}.$$

These results allow for inferences to be made not only on both sets of regression parameters but also on quantities associated with the baseline hazard function; see the results report in Section 5.

## 5 SIMULATION STUDIES

The performance of the proposed method is illustrated via two Monte Carlo experiments. The first simulation study evaluates the performance of our proposed MPL method for partly interval censored data with a comparison to the generalized odds rate mixture cure model proposed by Zhou et al,^17^ implemented in the GORCure R package, which includes the mixture cure Cox model as a special case. This method uses an expectation‐maximization (EM) algorithm with a gamma‐Poisson data augmentation for the regression parameters, and a spline approximation to a transformed cumulative hazard function. The second simulation study compares the EM based method of Reference 12 with our approach as the former is already implemented in the R package smcure. This method uses an EM algorithm for both Cox and logistic regression parameter estimation and a Breslow estimator for the baseline survival function. However, smcure can only be implemented to right‐censored survival datasets, so this simulation considers only right censoring.

### 5.1 Simulation study design and data generation

In order to generate event or censoring time(s) for individual i, we first computed the non‐cured fraction probability $\pi (z_{i})$ given by the following logistic model:

$$\pi (z_{i})=\frac{\exp(\beta_{0}+z_{i1}\beta_{1}+z_{i2}\beta_{2})}{1+\exp(\beta_{0}+z_{i1}\beta_{1}+z_{i2}\beta_{2})},$$

where $z_{i1}$ and $z_{i2}$ are logistic regression covariates. Note that the value of β controls the size of the cured fraction in the sample. Then, the cured indicator value $u_{i}$ was obtained according to

$$u_{i}=\left\{\begin{array}{cc} 0\; & \text{if}\;U_{i}>\pi (z_{i});\\ 1\; & \text{if}\;U_{i}\leq \pi (z_{i}), \end{array}\right.$$

where $U_{i}∼\text{unif}(0,1)$. Note that $u_{i}=1$ means individual i was in the non‐cured fraction (those who will experience the event of interest), and otherwise was in the cured fraction.

For $u_{i}=1$ in either study, we first simulated a follow‐up time $C_{i1}∼\text{unif}(0.5,\tau )$, where τ could be adjusted to control the extent of right censoring. Afterward, we simulated an event time $Y_{i}$ from a specified distribution. In Study 1, event times were drawn from one of three possible distributions (Weibull, exponential, and log‐logistic). In the smaller Study 2, only a Weibull distribution was considered. For $u_{i}=0$, we simulated a follow‐up time $C_{i2}∼\text{unif}(0.5,\tau )$, where τ has been defined before. Afterwards, an observed survival time, denoted by $T_{i}$, was simulated using the procedure described below.

For $u_{i}=0$, we took $T_{i}=C_{i2}$ and recorded it as a right censoring time. For $u_{i}=1$, $T_{i}$ was generated depending on Study 1 or Study 2. In Study 2 (right censoring), $T_{i}$ was simply generated using $T_{i}=\min\{Y_{i},C_{i1}\}$. In Study 1 (partly interval censoring), $T_{i}$ was generated based on a sequence of simulated “examination times” as described below. We first generated a number of “scheduled examinations” from a Poisson(8) (ie, mean 8) distribution, and denote this number by $d_{i}$. Next, we simulated $d_{i}$ uniform random numbers from unif(0.3,0.7), and denoted these numbers by $w_{i1},w_{i2},\ldots ,w_{id_{i}}$. Then, a series of “scheduled examination” times were then given by: $w_{i1},w_{i1}+w_{i2},\ldots ,\sum_{r=1}^{d_{i}}w_{ir}$. However, the follow‐up time $C_{i1}$ meant not all of these examination times might be used. In fact, when $C_{i1}$ was less than one of the examination times, the examination process would be terminated at $C_{i1}$ and the corresponding final examination times formed time intervals and we then checked if the simulated $Y_{i}$ fell into one of these time intervals. If yes, then $Y_{i}$ was interval censored and interval censoring times were given by the two end‐points of this interval. If no, $Y_{i}$ could be on the left or right of these intervals: if on the left then the minimum examination time was a left censoring time; if on the right then the maximum examination time was a right censoring time. Moreover, for an interval censoring, if the length of the interval was less than some δ then we recorded corresponding $Y_{i}$ as an event time rather than an interval‐censoring time. Details of all simulation scenarios considered can be found in Table 1 for Study 1 (partly interval censoring) and Table 2 for Study 2 (right censoring). We will report in this article the simulation results associated with the Weibull distribution with sample sizes n=200 and n=1000 and cured fractions sizes 0.2 and 0.6. Results for other distributions, the other sample size (n=50), and the other cured fraction size (0.05) can be found in the Supplementary Materials.

**TABLE 1:** Simulation study 1 (partly interval censoring) specifications, and the consequent cured and censoring proportions

	Scenario 1	Scenario 2	Scenario 3
Baseline hazard	$h_{0}(t)=3t^{2}$	$h_{0}(t)=t$	$h_{0}(t)=4.5t/(1+t^{2})$
Event times distribution	Weibull	Exponential	Log‐logistic
Sample sizes	n = 50, 200, 1000	n = 50, 200, 1000	n = 50, 200, 1000
Covariates	$x_{i}=[x_{i1},x_{i2}]$	$x_{i}=[x_{i1},x_{i2}]$	$x_{i}=[x_{i1},x_{i2}]$
	$z_{i}=[1,z_{i1},z_{i2}]$	$z_{i}=[1,z_{i1},z_{i2}]$	$z_{i}=[1,z_{i1},z_{i2}]$
	$x_{i1}∼\text{Bern}(0.5)$	$x_{i1}∼\text{Bern}(0.5)$	$x_{i1}∼\text{Bern}(0.5)$
	$x_{i2}∼N(0,1)$	$x_{i2}∼N(0,1)$	$x_{i2}∼N(0,1)$
	$z_{i1}∼\text{Bern}(0.5)$	$z_{i1}∼\text{Bern}(0.5)$	$z_{i1}∼\text{Bern}(0.5)$
	$z_{i2}∼N(0,1)$	$z_{i2}∼N(0,1)$	$z_{i2}∼N(0,1)$
Parameters	$\gamma_{1}=0.5,\gamma_{2}=1$	$\gamma_{1}=0.5,\gamma_{2}=1$	$\gamma_{1}=0.5,\gamma_{2}=1$
	$\beta_{0}=3,1.5,-0.5$	$\beta_{0}=1.5,-0.5$	$\beta_{0}=1.5,-0.5$
	$\beta_{1}=1,\beta_{2}=-0.5$	$\beta_{1}=1,\beta_{2}=-0.5$	$\beta_{1}=1,\beta_{2}=-0.5$
Cured fraction	1−π(z)=0.05,0.2,0.6	1−π(z)=0.2,0.6	1−π(z)=0.2,0.6
Event proportion in uncured fraction	$\pi^{E}=0.65,0.38,0.0$	$\pi^{E}=0.65,0.38,0.0$	$\pi^{E}=0.65,0.38,0.0$

**TABLE 2:** Simulation study 2 (right censoring) specifications, and the consequent cured and censoring proportions

	Scenario 1
Baseline hazard	$h_{0}(t)=3t^{2}$
Event times distribution	Weibull
Sample sizes	n = 200, 1000
Covariates	$x_{i}=[x_{i1},x_{i2}]$
	$z_{i}=[1,x_{i1},x_{i2}]$
	$x_{i1}∼\text{Bern}(0.5)$
	$x_{i2}∼U(0,1)$
Parameters	$\gamma_{1}=-0.5,\gamma_{2}=1$
	$\beta_{0}=1,-0.5$
	$\beta_{1}=0.5,\beta_{2}=-0.5$
Cured fraction	1−π(z)=0.27,0.61
Event proportion in uncured fraction	$\pi^{E}=0.85,0.5$

We adopted cubic M‐splines (with some $n_{\kappa }$ knots) to approximate the baseline hazard function. Define a and b as the minimum and maximum observed survival times, respectively. The observed survival times included interval, left, and right censoring times. External knots for the M‐splines were placed at a and b. Define c and d as the respective minimum and maximum of a set of times (called pseudo times) consisting of event times, the mid‐point of any left censoring intervals, and the mid‐point of any interval censoring intervals. The internal knots were placed at equal quantiles across the 5th and 95th percentiles of these pseudo times between c and d. Following Reference 23, we calculated the number of (internal) knots using a rough guideline of the cubic root of the number non‐right censored individuals. Ma et al^23^ remarked that the MPL method is fairly robust (due to the penalty function) to the number of knots as long as the smoothing parameter λ is appropriate. The smoothing parameter was selected automatically using the marginal likelihood function laid out above in Section 3.

Note that we have presented results based on estimates of the marginal (non‐cured fraction only) baseline survival function rather than the marginal baseline hazard function, as quantities of the marginal baseline survival function are more readily available in the GORCure and smcure packages. Computing the marginal baseline survival function and associated asymptotic and Monte Carlo standard errors for the MPL method is straightforward. At time t, the MPL estimate of $S_{0}(t)$ can be computed by taking $\exp(-\Psi {\left(t\right)}^{T}\hat{\theta })$, where Ψ(t) denotes a vector of I‐spline values at t. The asymptotic variance estimate can be produced using the delta method, such that $\mathrm{Var}({Ŝ}_{0}(t))={\left[\Psi {\left(t\right)}^{⊤}{Ŝ}_{0}(t)\right]}^{⊤}\text{Cov}(\hat{\theta })[\Psi {\left(t\right)}^{⊤}{Ŝ}_{0}(t)]$, where $\Psi {\left(t\right)}^{⊤}{Ŝ}_{0}(t)$ is the first derivative of ${Ŝ}_{0}(t)$ with respect to $\hat{\theta }$. The Monte Carlo variance can be obtained simply by replacing $\text{Cov}(\hat{\theta })$ with the Monte Carlo covariance matrix for θ in the equation for $\mathrm{Var}({Ŝ}_{0}(t))$.

### 5.2 Study 1 (partly interval censoring) results

Table 3 shows the results for the estimation of β and γ for Study 1 (partly interval censoring) for sample sizes n=200 and n=1000, for the MPL method and the GOR method, using a Weibull distribution for the baseline hazard. It includes, for each parameter, the absolute bias, the relative bias (in brackets beneath the absolute bias), the asymptotic standard error estimate, the Monte Carlo standard error estimate (in brackets beneath the asymptotic standard error), and the asymptotic 95% coverage probability. The MPL method appears to perform reasonably in a variety of the scenarios considered, with small biases, good agreement between the asymptotic and Monte Carlo standard errors, and reasonable coverage probabilities. In particular, when the sample size is small, the MPL estimates for the proportional hazards regression parameters ($\gamma_{1}$ and $\gamma_{2}$) consistently have smaller biases than the GOR estimates. This is especially noticeable in the 1−π(z)=0.6 scenarios, that is, when there is a larger number of cured individuals in the sample. Additionally, across all scenarios, the GOR estimate of the $\beta_{0}$ has a large negative bias and has very low coverage probabilities, while the MPL estimate of that parameter is reasonable. This equates to an overestimation of the cured fraction size by the GOR method.

**TABLE 3:** Study 1 (partly interval censoring): Cox and logistic regression parameters for n=200 and n=1000 for Scenario 1 (Weibull baseline)

			1−π(z)=0.2									1−π(z)=0.6								
			$\pi^{E}=0.65$			$\pi^{E}=0.38$			$\pi^{E}=0.0$			$\pi^{E}=0.65$			$\pi^{E}=0.38$			$\pi^{E}=0.0$		
			Bias	SE	CP	Bias	SE	CP	Bias	SE	CP	Bias	SE	CP	Bias	SE	CP	Bias	SE	CP
n=200	$\beta_{0}$	MPL	0.049	0.211	0.96	0.070	0.214	0.96	0.025	0.210	0.97	−0.005	0.160	0.95	−0.021	0.160	0.95	−0.001	0.159	0.95
			(0.033)	(0.208)		(0.046)	(0.224)		(0.017)	(0.210)		(0.010)	(0.163)		(0.041)	(0.160)		(0.001)	(0.163)	
		GOR	−0.457	0.237	0.50	−0.468	0.238	0.49	−0.497	0.242	0.45	−0.513	0.235	0.42	−0.497	0.234	0.42	−0.510	0.214	0.01
			(0.305)	(0.227)		(0.312)	(0.236)		(0.332)	(0.254)		(1.026)	(0.242)		(0.995)	(0.207)		(1.019)	(0.267)	
	$\beta_{1}$	MPL	0.033	0.407	0.96	0.051	0.413	0.94	0.043	0.406	0.95	0.044	0.321	0.95	0.026	0.320	0.96	0.017	0.320	0.96
			(0.033)	(0.411)		(0.051)	(0.461)		(0.043)	(0.413)		(0.044)	(0.335)		(0.026)	(0.329)		(0.017)	(0.315)	
		GOR	−0.014	0.394	0.94	0.053	0.397	0.97	0.045	0.406	0.95	0.021	0.316	0.91	−0.021	0.314	0.96	0.018	0.287	0.86
			(0.014)	(0.422)		(0.053)	(0.381)		(0.045)	(0.419)		(0.021)	(0.336)		(0.021)	(0.308)		(0.018)	(0.316)	
	$\beta_{2}$	MPL	−0.020	0.200	0.95	−0.006	0.201	0.95	−0.013	0.200	0.93	−0.022	0.169	0.95	−0.014	0.167	0.94	−0.011	0.167	0.96
			(0.041)	(0.206)		(0.013)	(0.201)		(0.026)	(0.212)		(0.044)	(0.170)		(0.029)	(0.170)		(0.022)	(0.172)	
		GOR	−0.010	0.197	0.95	−0.004	0.197	0.94	−0.014	0.202	0.94	−0.014	0.166	0.95	−0.010	0.165	0.97	−0.012	0.151	0.86
			(0.019)	(0.203)		(−0.008)	(0.209)		(0.029)	(0.213)		(0.027)	(0.174)		(0.019)	(0.144)		(0.024)	(0.175)	
	$\gamma_{1}$	MPL	−0.004	0.165	0.95	−0.010	0.165	0.96	0.003	0.167	0.95	−0.008	0.238	0.92	−0.008	0.239	0.93	0.001	0.237	0.94
			(0.008)	(0.167)		(0.020)	(0.162)		(0.006)	(0.174)		(0.016)	(0.254)		(0.015)	(0.252)		(0.001)	(0.250)	
		GOR	0.033	0.172	0.96	0.056	0.176	0.95	−0.021	0.180	0.95	0.010	0.247	0.94	0.008	0.256	0.96	−0.453	0.250	0.71
			(0.066)	(0.174)		(0.112)	(0.174)		(0.041)	(0.183)		(0.020)	(0.255)		(0.016)	(0.247)		(0.907)	(0.171)	
	$\gamma_{2}$	MPL	−0.009	0.100	0.92	−0.002	0.101	0.92	−0.004	0.101	0.93	−0.002	0.142	0.90	0.001	0.142	0.89	0.002	0.140	0.88
			(0.009)	(0.108)		(0.002)	(0.112)		(0.003)	(0.109)		(0.002)	(0.162)		(0.001)	(0.168)		(0.002)	(0.169)	
		GOR	0.050	0.113	0.94	0.043	0.118	0.96	0.032	0.117	0.96	−0.017	0.160	0.93	0.009	0.164	0.97	−0.902	0.165	0.07
			(0.050)	(0.119)		(0.043)	(0.119)		(0.032)	(0.112)		(0.017)	(0.227)		(0.009)	(0.164)		(0.902)	(0.325)	
n=1000	$\beta_{0}$	MPL	0.018	0.092	0.94	0.017	0.092	0.96	0.014	0.092	0.94	−0.004	0.071	0.94	0.005	0.070	0.96	−0.001	0.071	0.95
			(0.012)	(0.094)		(0.011)	(0.089)		(0.009)	(0.092)		(0.007)	(0.074)		(0.009)	(0.068)		(0.001)	(0.072)	
		GOR	−0.486	0.105	0.02	−0.491	0.104	0.01	−0.484	0.107	0.01	−0.494	0.104	0.00	−0.507	0.104	0.00	−0.504	0.104	0.00
			(0.324)	(0.109)		(0.327)	(0.108)		(0.323)	(0.104)		(0.987)	(0.096)		(1.013)	(0.100)		(1.008)	(0.110)	
	$\beta_{1}$	MPL	0.019	0.177	0.95	0.010	0.177	0.95	−0.006	0.177	0.95	0.012	0.141	0.95	0.005	0.141	0.96	0.001	0.141	0.95
			(0.019)	(0.174)		(0.010)	(0.176)		(0.006)	(0.178)		(0.012)	(0.137)		(0.005)	(0.133)		(0.001)	(0.141)	
		GOR	0.002	0.172	0.95	0.002	0.172	0.94	0.008	0.176	0.95	0.001	0.139	0.95	0.001	0.139	0.96	−0.002	0.139	0.93
			(0.002)	(0.167)		(0.002)	(0.174)		(0.008)	(0.178)		(0.001)	(0.133)		(0.001)	(0.138)		(0.002)	(0.141)	
	$\beta_{2}$	MPL	−0.008	0.088	0.95	−0.003	0.088	0.95	0.001	0.088	0.95	−0.002	0.074	0.92	−0.005	0.074	0.95	0.001	0.073	0.95
			(0.015)	(0.090)		(−0.006)	(0.088)		(0.001)	(0.090)		(0.004)	(0.079)		(0.009)	(0.075)		(0.001)	(0.073)	
		GOR	−0.009	0.086	0.93	0.005	0.086	0.98	0.002	0.088	0.94	−0.010	0.073	0.96	−0.008	0.073	0.97	0.001	0.073	0.93
			(0.018)	(0.092)		(0.011)	(0.082)		(0.003)	(0.089)		(0.019)	(0.074)		(0.015)	(0.065)		(0.001)	(0.073)	
	$\gamma_{1}$	MPL	0.003	0.075	0.94	−0.005	0.075	0.95	−0.006	0.076	0.97	0.002	0.107	0.95	−0.001	0.107	0.94	−0.010	0.108	0.95
			(0.006)	(0.078)		(0.009)	(0.078)		(0.013)	(0.075)		(0.003)	(0.110)		(0.001)	(0.110)		(0.019)	(0.109)	
		GOR	0.005	0.075	0.95	0.001	0.076	0.93	−0.001	0.077	0.97	0.001	0.106	0.95	0.011	0.109	0.93	−0.347	0.115	0.29
			(0.009)	(0.077)		(0.002)	(0.082)		(0.001)	(0.076)		(0.001)	(0.104)		(0.022)	(0.107)		(0.693)	(0.241)	
	$\gamma_{2}$	MPL	−0.008	0.046	0.93	−0.007	0.046	0.94	−0.005	0.046	0.92	−0.008	0.063	0.91	−0.002	0.063	0.92	−0.013	0.064	0.92
			(0.008)	(0.049)		(0.007)	(0.046)		(0.005)	(0.050)		(0.008)	(0.072)		(0.002)	(0.073)		(0.018)	(0.071)	
		GOR	0.011	0.048	0.97	0.001	0.050	0.96	0.007	0.050	0.95	−0.014	0.067	0.93	−0.001	0.069	0.97	−0.694	0.079	0.28
			(0.011)	(0.045)		(0.001)	(0.051)		(0.007)	(0.051)		(0.014)	(0.072)		(0.001)	(0.063)		(0.694)	(0.468)	

Abbreviations: CP, coverage probability; SE, standard error.

Table 4 shows the results for the estimation of the baseline survival function for Study 1 (partly interval censoring) for sample sizes n=200 and n=1000, for the MPL method and the GOR method, using a Weibull distribution for the baseline hazard. For the MPL method it reports the bias, asymptotic and (Monte Carlo) standard errors, and asymptotic and (Monte Carlo) 95% coverage probabilities. For the GOR method, it reports the bias, (Monte Carlo) standard errors and (Monte Carlo) 95% coverage probabilities, as asymptotic estimates for the standard error are unavailable from the GORCure package. The ability to make fast and efficient inferences on survival model quantities using asymptotic standard errors can be considered as a key strength of the proposed MPL method when compared with the existing alternatives. The estimated survival function was evaluated at three time points, $t_{1}$, $t_{2}$, and $t_{3}$, corresponding to the first, second, and third quartile of the observed survival times, excluding 0 and ∞. Across all scenarios, the bias for the MPL estimate of the baseline survival function was small. There is a fair agreement between the asymptotic and Monte Carlo standard error estimates for the MPL method across the scenarios. We note that in some instances, the coverage probabilities produced by the MPL method are low; specifically, this tends to occur at the later time point $t_{3}$ where event times are sparser. The baseline survival function estimates from the GOR method tend to have larger biases than the MPL estimates, particularly when $\pi^{E}=0$. The Monte Carlo standard errors for the GOR estimates are generally larger than both the asymptotic and Monte Carlo standard error estimates from the MPL method, indicating that the MPL method is less variable.

**TABLE 4:** Study 1 (partly interval censoring): Baseline survival function estimation for n=200 and n=1000 for Scenario 1 (Weibull baseline)

			1−π(z)=0.2									1−π(z)=0.6								
			$\pi^{E}=0.65$			$\pi^{E}=0.38$			$\pi^{E}=0.0$			$\pi^{E}=0.65$			$\pi^{E}=0.38$			$\pi^{E}=0.0$		
			Bias	SE	CP	Bias	SE	CP	Bias	SE	CP	Bias	SE	CP	Bias	SE	CP	Bias	SE	CP
n=200	$t_{1}$	MPL	−0.005	0.029	0.93	−0.002	0.029	0.93	−0.007	0.029	0.93	−0.002	0.037	0.92	−0.007	0.036	0.90	−0.007	0.036	0.90
				(0.033)	(0.95)		(0.039)	(0.98)		(0.040)	(0.96)		(0.038)	(0.90)		(0.047)	(0.93)		(0.053)	(0.96)
		GOR	−0.010	‐	‐	−0.033	‐	‐	0.066	‐	‐	0.004	‐	‐	−0.010	‐	‐	0.397	‐	‐
				(0.103)	(0.86)		(0.102)	(0.90)		(0.071)	(0.96)		(0.107)	(0.90)		(0.100)	(0.88)		(1.100)	(0.89)
	$t_{2}$	MPL	0.002	0.020	0.93	0.001	0.021	0.93	0.002	0.020	0.93	−0.001	0.038	0.90	0.001	0.036	0.90	0.003	0.036	0.92
				(0.016)	(0.89)		(0.024)	(0.98)		(0.031)	(0.97)		(0.029)	(0.90)		(0.046)	(0.96)		(0.129)	(1.00)
		GOR	−0.026	‐	‐	0.048	‐	‐	0.082	‐	‐	−0.027	‐	‐	0.050	‐	‐	−0.253	‐	‐
				(0.113)	(0.88)		(0.128)	(0.87)		(0.125)	(1.00)		(0.150)	(0.86)		(0.157)	(0.89)		(0.953)	(0.89)
	$t_{3}$	MPL	0.001	0.002	0.89	0.001	0.002	0.87	0.001	0.002	0.88	0.001	0.007	0.83	0.002	0.006	0.84	0.001	0.006	0.86
				(0.001)	(0.84)		(0.001)	(0.88)		(0.003)	(0.88)		(0.004)	(0.82)		(0.007)	(0.83)		(0.025)	(1.00)
		GOR	−0.005	‐	‐	−0.006	‐	‐	0.009	‐	‐	−0.014	‐	‐	−0.018	‐	‐	−0.233	‐	‐
				(0.016)	(0.94)		(0.018)	(0.91)		(0.021)	(0.94)		(0.043)	(0.88)		(0.051)	(0.90)		(0.889)	(0.90)
n=1000	$t_{1}$	MPL	−0.002	0.024	0.98	−0.001	0.211	0.99	−0.001	0.038	0.94	−0.002	0.118	0.95	−0.001	0.020	0.92	−0.003	0.148	0.96
(0.018)				(0.95)	(0.031)		(1.00)	(0.030)		(1.00)	(0.030)		(1.00)	(0.037)		(1.00)	(0.025)		(0.98)
GOR		0.026	‐	‐	0.013	‐	‐	0.082	‐	‐	0.015	‐	‐	−0.003	‐	‐	−0.043	‐	‐
			(0.113)	(0.87)		(0.114)	(0.91)		(0.063)	(0.95)		(0.104)	(0.88)		(0.107)	(0.91)		(0.402)	(0.99)
$t_{2}$	MPL	0.001	0.005	0.92	0.001	0.005	0.93	0.001	0.006	0.93	0.001	0.010	0.91	−0.001	0.010	0.93	0.001	0.011	0.92
			(0.003)	(0.95)		(0.005)	(0.99)		(0.006)	(0.97)		(0.006)	(0.92)		(0.013)	(0.97)		(0.008)	(0.94)
	GOR	0.001	‐	‐	−0.009	‐	‐	0.047	‐	‐	−0.006	‐	‐	−0.019	‐	‐	0.040	‐	‐
			(0.036)	(0.83)		(0.052)	(0.93)		(0.053)	(0.98)		(0.070)	(0.90)		(0.069)	(0.86)		(0.343)	(0.99)
$t_{3}$	MPL	0.001	0.001	0.93	0.001	0.001	0.94	0.001	0.001	0.93	0.001	0.001	0.87	0.001	0.001	0.86	0.001	0.001	0.91
			(0.001)	(0.89)		(0.001)	(0.92)		(0.001)	(0.91)		(0.001)	(0.88)		(0.001)	(0.89)		(0.007)	(0.88)
	GOR	0.001	‐	‐	0.001	‐	‐	0.001	‐	‐	0.001	‐	‐	0.001	‐	‐	−0.012	‐	‐
			(0.001)	(0.94)		(0.002)	(0.96)		(0.002)	(0.95)		(0.005)	(0.97)		(0.003)	(0.93)		(0.322)	(0.99)

Abbreviations: CP, coverage probability; SE, standard error.

We have included in the Supplementary Materials some additional simulation study results for partly interval censored data. These include results from scenarios where we have used different distributions for the baseline hazard function (namely, exponential and log‐logistic), scenarios where the sample size was very small (n=50), and scenarios where the cured fraction was very small (approximately 5% of the sample). The exponential and log‐logistic baseline hazard distribution scenarios have generally produced comparable results to those discussed above. When the sample size is very small, both methods tend to produce larger biases in the regression parameter estimates. However, biases and coverage probabilities appear to be more reasonable for the MPL estimates, particularly for the proportional hazards regression parameter estimates ($\gamma_{1}$ and $\gamma_{2}$) when the cured fraction in the sample is larger. When the cured fraction was very small, the MPL estimates of regression parameters and the baseline survival function were still reasonable.

### 5.3 Study 2 (right censoring) results

Table 5 exhibits the results for the estimation of β and γ for Study 2 (right censoring) with n=200 and n=1000 for both the MPL and EM methods. It includes, for each parameter, the absolute bias, relative bias (in brackets below absolute bias), the average standard error (asymptotic and bootstrap for, respectively, MPL and EM), the Monte Carlo standard error (in brackets below average SE), and the 95% coverage probability. Note that the EM method in the smcure R package does not provide directly an asymptotic standard error, instead it provides a computationally intensive bootstrapping standard error.

**TABLE 5:** Study 2 (right censoring): Cox and logistic regression parameters for n=200 and n=1000 for MPL and EM methods

			1−π(z)=0.27						1−π(z)=0.61					
			$\pi^{E}=0.85$			$\pi^{E}=0.5$			$\pi^{E}=0.85$			$\pi^{E}=0.5$		
			Bias	SE	CP	Bias	SE	CP	Bias	SE	CP	Bias	SE	CP
n=200	$\beta_{0}$	MPL	0.046	0.251	0.93	0.080	0.795	0.81	−0.008	0.172	0.94	0.096	0.407	0.94
			(0.046)	(0.250)		(0.080)	(0.836)		(0.016)	(0.181)		(0.192)	(0.394)	
		EM	0.029	0.414	0.95	−0.910	0.336	0.28	−0.262	0.194	0.70	−0.265	0.419	0.89
			(0.029)	(0.430)		(0.910)	(0.430)		(0.524)	(0.253)		(0.530)	(0.469)	
	$\beta_{1}$	MPL	0.042	0.500	0.95	−0.329	0.897	0.94	0.018	0.344	0.96	0.128	0.462	0.94
			(0.084)	(0.462)		(0.658)	(1.024)		(0.035)	(0.360)		(0.256)	(0.469)	
		EM	0.019	0.415	0.96	−0.424	0.546	0.90	0.005	0.354	0.96	0.067	0.405	0.95
			(0.038)	(0.394)		(0.847)	(0.503)		(0.010)	(0.358)		(0.134)	(0.416)	
	$\beta_{2}$	MPL	−0.050	0.668	0.95	0.373	0.608	0.68	−0.024	0.193	0.94	−0.093	0.718	0.96
			(0.100)	(0.714)		(0.747)	(1.231)		(0.048)	(0.200)		(0.186)	(0.731)
		EM	−0.017	0.695	0.95	0.673	0.353	0.44	−0.001	0.199	0.94	0.060	0.702	0.97
			(0.034)	(0.684)		(1.347)	(0.303)		(0.003)	(0.198)		(0.120)	(0.677)	
	$\gamma_{1}$	MPL	−0.013	0.191	0.93	0.164	0.795	0.91	−0.037	0.172	0.76	0.049	0.401	0.93
			(0.026)	(0.199)		(0.329)	(0.523)		(0.074)	(0.288)		(0.098)	(0.392)
		EM	−0.020	0.207	0.94	0.157	0.395	0.92	−0.029	0.315	0.96	−0.021	0.366	0.96
			(0.040)	(0.202)		(0.315)	(0.402)		(0.059)	(0.290)		(0.042)	(0.357)	
	$\gamma_{2}$	MPL	0.019	0.332	0.93	−0.193	0.897	0.99	0.015	0.344	1.00	0.103	0.694	0.92
			(0.019)	(0.358)		(0.193)	(0.480)		(0.015)	(0.176)		(0.206)	(0.694)
		EM	0.028	0.365	0.95	−0.280	0.269	0.83	0.010	0.200	0.97	0.033	0.649	0.95
			(0.028)	(0.363)		(0.280)	(0.309)		(0.010)	(0.180)		(0.066)	(0.640)	
n=1000	$\beta_{0}$	MPL	0.013	0.084	0.95	0.132	0.325	0.92	−0.004	0.076	0.94	0.037	0.105	0.94
			(0.013)	(0.085)		(0.132)	(0.384)		(0.008)	(0.079)		(0.074)	(0.134)	
		EM	0.001	0.171	0.94	0.803	0.197	0.02	−0.260	0.082	0.14	0.253	0.174	0.66
			(0.001)	(0.179)		(0.803)	(0.206)		(0.520)	(0.109)		(0.506)	(0.195)	
	$\beta_{1}$	MPL	0.016	0.167	0.93	0.050	0.342	0.91	0.010	0.152	0.94	0.007	0.174	0.96
			(0.032)	(0.174)		(0.099)	(0.418)		(0.020)	(0.153)		(0.014)	(0.175)
		EM	0.014	0.167	0.93	−0.342	0.218	0.57	0.007	0.152	0.92	−0.007	0.167	0.96
			(0.028)	(0.174)		(0.683)	(0.255)		(0.015)	(0.154)		(0.014)	(0.159)	
	$\beta_{2}$	MPL	0.001	0.287	0.95	−0.041	0.216	0.89	−0.017	0.084	0.94	0.019	0.293	0.94
			(0.002)	(0.285)		(0.083)	(0.339)		(0.034)	(0.091)		(0.038)	(0.301)	
		EM	0.006	0.288	0.95	0.545	0.125	0.10	−0.011	0.085	0.94	0.026	0.291	0.93
			(0.012)	(0.285)		(1.090)	(0.174)		(0.021)	(0.091)		(0.052)	(0.296)	
	$\gamma_{1}$	MPL	0.009	0.086	0.94	0.004	0.325	1.00	0.013	0.076	0.74	−0.008	0.159	0.96
			(0.018)	(0.088)		(0.008)	(0.157)		(0.026)	(0.134)		(0.016)	(0.145)	
		EM	0.015	0.154	0.93	0.091	0.159	0.90	0.005	0.128	0.94	−0.003	0.259	0.97
			(0.030)	(0.090)		(0.181)	(0.159)		(0.010)	(0.137)		(0.006)	(0.143)	
	$\gamma_{2}$	MPL	0.002	0.149	0.93	0.002	0.339	1.00	−0.007	0.152	1.00	0.025	0.276	0.96
			(0.002)	(0.158)		(0.002)	(0.118)		(0.007)	(0.075)		(0.050)	(0.258)	
		EM	0.015	0.154	0.93	−0.173	0.099	0.56	0.005	0.080	0.95	−0.003	0.259	0.97
			(0.015)	(0.161)		(0.173)	(0.119)		(0.005)	(0.078)		(0.006)	(0.248)	

Abbreviations: CP, coverage probability; SE, standard error.

The results from Table 5 indicate the two methods appear largely equivalent under the scenario with cured fraction 1−π(z)=0.7 and $\pi^{E}=0.85$, with both methods producing small biases in the parameter estimates, having good agreement between the estimated (asymptotic for MPL and bootstrap for EM) and Monte Carlo standard errors, and giving reasonable coverage probabilities. When the cured fraction is 1−π(z)=0.7 but $\pi^{E}=0.5$, the bias for most parameter estimates is fairly large for both methods, although it is generally larger for the EM estimates compared to the MPL estimates, and the EM coverage probabilities for this scenario are particularly low. However, when the sample size is large, the MPL estimates for the proportional hazards regression parameters ($\gamma_{1}$ and $\gamma_{2}$) have small biases, while the equivalent estimates for the EM method produce very large biases. However, for these MPL estimates the coverage probabilities are high, reflecting the larger asymptotic SE estimates compared to the Monte Carlo estimates. When the simulated data contained a larger cured fraction, both methods similarly yield little bias in the parameter estimates with the exception of the estimate for $\beta_{0}$. The EM estimate of $\beta_{0}$ has a large negative bias and that is less severe in the MPL estimate. We observe from the results that in general the MPL standard error matches well the Monte Carlo standard error for the higher censoring scenario, but less well for the scenario with less censoring, especially for the proportional hazards parameters $\gamma_{1}$ and $\gamma_{2}$. For a large sample size (ie, n=1000), the asymptotic standard error produced by the MPL method are generally close to with the bootstrap standard errors from the EM method, except $\beta_{0}$. However, computations of MPL asymptotic standard errors are much faster than the EM bootstrap standard errors.

Table 6 reports the biases, Monte Carlo standard errors and Monte Carlo coverage probabilities for the estimated baseline survival function ${Ŝ}_{0}(t)$ from both the MPL and EM methods. The estimated survival function was evaluated at three time points, $t_{1}$, $t_{2}$, and $t_{3}$, corresponding to the first, second, and third quartile of the observed event times, excluding 0 and ∞. Note that for comparison purposes only the Monte Carlo standard errors and Monte Carlo coverage probabilities are reported here for both MPL and EM since the smcure package does not provide asymptotic nor bootstrapped standard error estimates for the baseline survival function. From Table 6, it is clear that the MPL method provides better estimates of the survival function in all scenarios than the EM method. MPL gives smaller biases across all the selected time points regardless of scenarios and samples sizes. Also, MPL provides smaller standard errors and better coverage probabilities than EM.

**TABLE 6:** Study 2 (right censoring): Baseline survival function estimation for n=200 and n=1000 for MPL and EM estimation methods

			1−π(z)=0.2						1−π(z)=0.6					
			$\pi^{E}=0.65$			$\pi^{E}=0.38$			$\pi^{E}=0.65$			$\pi^{E}=0.38$		
			Bias	SE	CP	Bias	SE	CP	Bias	SE	CP	Bias	SE	CP
n=200	$t_{1}$	MPL	0.002	0.032	0.94	−0.003	0.015	0.95	−0.002	0.100	1.00	0.013	0.052	0.94
		EM	0.049	0.054	0.86	−0.018	0.021	0.85	−0.053	0.120	0.99	0.044	0.085	0.92
	$t_{2}$	MPL	0.002	0.039	0.95	−0.014	0.056	0.95	−0.004	0.126	1.00	0.020	0.074	0.92
		EM	0.084	0.080	0.81	−0.106	0.071	0.67	−0.056	0.119	0.98	0.075	0.132	0.91
	$t_{3}$	MPL	0.003	0.036	0.95	−0.016	0.107	0.94	0.001	0.019	0.98	0.027	0.095	0.94
		EM	0.088	0.083	0.81	−0.197	0.109	0.55	−0.005	0.013	0.93	0.090	0.147	0.91
n=1000	$t_{1}$	MPL	−0.001	0.014	0.95	−0.001	0.005	0.94	−0.002	0.088	1.00	0.006	0.023	0.96
		EM	0.049	0.024	0.45	0.017	0.010	0.61	−0.070	0.102	1.00	0.050	0.034	0.68
	$t_{2}$	MPL	−0.001	0.017	0.96	0.001	0.021	0.96	0.002	0.048	1.00	0.011	0.036	0.96
		EM	0.084	0.037	0.38	−0.104	0.033	0.11	−0.027	0.032	0.93	0.084	0.056	0.69
	$t_{3}$	MPL	−0.001	0.016	0.95	0.009	0.047	0.94	0.001	0.001	0.99	0.015	0.044	0.96
		EM	0.091	0.039	0.34	−0.194	0.049	0.03	−0.001	0.001	0.96	0.091	0.063	0.73

Abbreviations: CP, coverage probability; SE, standard error.

## 6 APPLICATION TO MELANOMA RECURRENCE DATA

Our new method was applied to the real dataset introduced in Section 1. Data from a cohort of 2968 patients diagnosed with thin melanoma (defined as patients diagnosed with a Breslow thickness ≤1 mm) between January 1992 and December 2014 were extracted from the prospectively maintained research database at the Melanoma Institute Australia (MIA). Information collected included baseline characteristics (age, sex, and body site of lesion), pathological factors (Breslow thickness, ulceration, and mitoses count), date of follow‐up visit, date of diagnosis of first recurrence (either local, regional, or distant), and survival status of patient at last contact. All patients with known date of recurrence or last survival status were analyzed. All patients had given consent for use of their de‐identified information for research purposes. Ethical approval was provided by the Sydney Local Health District Ethics Committee.

The primary clinical outcome was time to first melanoma recurrence calculated from the date of the initial primary diagnosis. Patients who experienced melanoma recurrence were subject to interval censoring, as the exact time of recurrence was unknown. The time interval in which the recurrence occurred was denoted $\left(t_{n},t_{r}\right)$ where $t_{n}$ was the preceding follow‐up visit date of the recurrence, also called the last known recurrence free time, and $t_{r}$ was the date recurrence was first diagnosed. Some patients did not have recorded values of $t_{n}$ and $t_{r}$, but had a status at their last follow up of either “alive with melanoma” or “dead, melanoma,” indicating that they had experienced a tumor recurrence at some point and thus left‐censored. These patients therefore had a left censoring interval of (0, $t_{f}-t_{d}$), where $t_{d}$ and $t_{f}$ denote, respectively, the starting and last follow‐up time. All other patients were right‐censored at the time of their last follow up.

Six categorical covariates were considered in this analysis and coded as: Breslow thickness (≤0.8 mm; >0.8 mm), tumor ulceration (yes; no), age group (<50; ≥50), sex (male; female), tumor mitoses (yes; no), and site of tumor (arm; head & neck; leg; trunk). Thin melanoma recurrence was rare, with only 6.69% of the sample having experienced the event at some point during the follow‐up time. The remaining individuals were right‐censored. This prevalence of right‐censored observations suggested that there was likely a cured fraction. A mixture cure Cox model was fitted with all six covariates in the latency model and the incidence model. The incidence and latency models were then reduced using backwards step‐wise selection with P‐values, until all covariates left in the model were significant at the 5% level.

Table 7 shows the odds ratios (from the incidence logistic regression model) and hazard ratios (from the latency proportional hazards model), 95% confidence intervals (CIs) and P‐values after implementing our proposed method. Results from the incidence model indicate that the odds of thin melanoma recurrence increase significantly in patients with Breslow thickness >0.8 mm, with mitoses, or who are male. Conversely, the odds of thin melanoma recurrence are significantly lower in patients who had a tumor on the leg or the trunk, compared to on the arm (the reference category). The most striking feature of the latency model for non‐cured patients was that Breslow thickness was not significantly associated with recurrence. Tumor ulceration and having a tumor on the head & neck, the leg or the trunk instead of the arm significantly increased the risk of melanoma recurrence. Sex was also significantly associated with risk of recurrence, with males in the non‐cured population having a significantly lower risk of melanoma recurrence than females. Age was not significant in either the incidence or the latency model.

**TABLE 7:** Model fitting results for the thin melanoma data

	MPL mixture cure model			GOR mixture cure model			Standard Cox model		
Covariate	OR/HR	95% CI	P‐value	OR/HR	95% CI	P‐value	HR	95% CI	P‐value
*Incidence model*									
Breslow thickness: >0.8 mm	1.98	(1.12, 3.48)	0.019	‐	‐	‐	‐	‐	‐
Ulceration: Yes	‐	‐	‐	3.01	(2.34, 3.88)	0.015	‐	‐	‐
Sex: Male	7.57	(3.76, 15.22)	<0.001	‐	‐	‐	‐	‐	‐
Mitoses: Yes	2.86	(1.66, 4.95)	0.001	‐	‐	‐	‐	‐	‐
Body site: Leg	0.28	(0.12, 0.62)	0.002	‐	‐	‐	‐	‐	‐
Body site: Trunk	‐	‐	‐	1.93	(1.27, 2.95)	0.002	‐	‐	‐
*Latency model*									
Breslow thickness: >0.8 mm	‐	‐	‐	2.24	(1.30, 3.86)	0.004	1.59	(1.05, 2.42)	0.029
Ulceration: Yes	2.38	(1.20, 4.73)	0.014	‐	‐	‐	2.38	(1.14, 4.97)	0.022
Sex: Male	0.15	(0.09, 0.28)	<0.001	‐	‐	‐	‐	‐	‐
Mitoses: Yes	‐	‐	‐	3.29	(1.95, 5.54)	<0.001	2.25	(1.46, 3.47)	<0.001
Body site: Head & Neck	2.80	(1.34, 5.86)	0.006	2.92	(1.57, 5.44)	<0.001	2.13	(1.23, 3.66)	0.007
Body site: Leg	4.98	(1.94, 12.82)	<0.001	‐	‐	‐	‐	‐	‐
Body site: Trunk	2.46	(1.34, 4.48)	0.003	‐	‐	‐	1.89	(1.25, 2.86)	0.003

Abbreviations: CI, confidence interval; HR, hazard ratio; OR, odds ratio.

Interpretation of the outputs of a mixture cure model demands some extra attention. Particularly, notice should be paid to the fact that the latency model is a conditional model, only relevant to the non‐cured sub‐population. For example, the latency model of this melanoma example suggests that, if we consider at the non‐cured (ie, melanoma recurrence) sub‐population, males have lower risk of recurrence than females. However, the results from the incidence model suggest males have higher odds of being classified into the non‐cured sub‐population. These two interpretations of sex in the incidence and latency model do not contradict each other. Corbière et al^19^ similarly found that some parameter estimates for the same covariates had different signs between the latency and the incidence models in their analysis of tonsil tumor recurrence data.

Table 7 also contains the results of fitting a proportional hazards mixture cure model using the GORCure package, and the Cox model results without a cured fraction (standard Cox model), fitted using the survivalMPL R package. The same six covariates were considered, and the same backwards step‐wise selection was carried out to select significant covariates. There are substantial differences between the MPL and GOR fitted models. There was no overlap between the MPL and GOR incidence models in terms of significant predictors of recurrence susceptibility, and only one (body site: head & neck) shared between the MPL and GOR latency model for risk of recurrence. Notably, according to the GOR model there was no significant difference between males and females for either incidence or latency, while sex was significant in both the latency and incidence MPL models. It is of interest to compare the results of the standard Cox model results with the latency model from the mixture cure model. For the standard Cox model, there was no significant difference between males and females in terms of melanoma recurrence risk, which contradicts the latency model result. Also, for the standard Cox model, Breslow thickness and mitoses are significant, but were not significant in the latency model. The magnitudes of the hazard ratios and the significance of the body site categories also differ between the latency sub‐model and the standard Cox model. Age group was not significant in either the latency or the standard Cox model.

**FIGURE 1:**
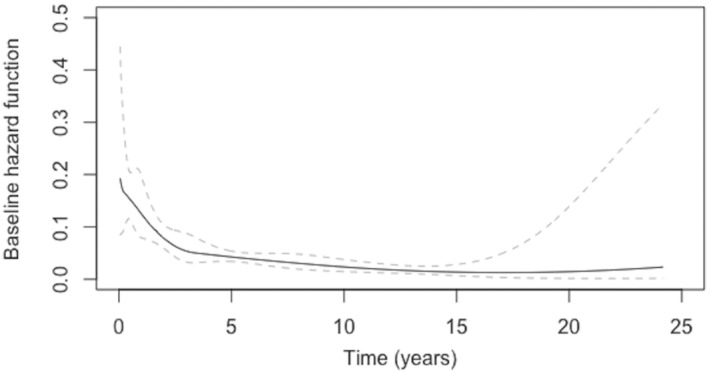
Estimated baseline hazard function for the non‐cured population (with point‐wise 95% confidence intervals)

**FIGURE 2:**
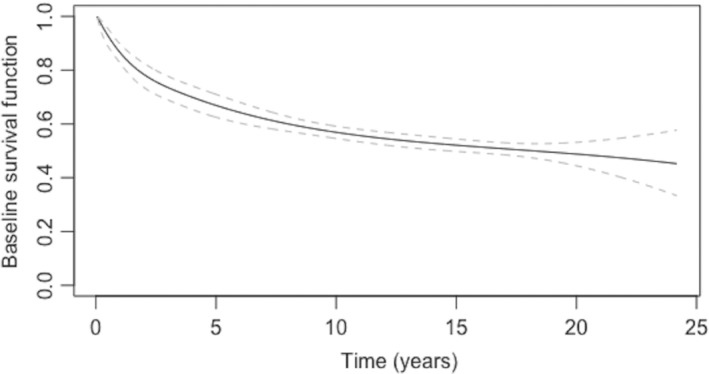
Estimated conditional baseline survival function for susceptible sub‐population (with point‐wise 95% confidence interval)

The estimate of the baseline hazard function for the melanoma recurrence sub‐population can be seen in Figure 1. There is a notably rapid drop in the risk of tumor recurrence over the first 3 to 4 years, after which the risk of recurrence decreases slowly. Between 20 and 25 years there appears to be a small increase in the risk, although the point‐wise confidence intervals are wide for this time period. Figure 2 exhibits the baseline survival function with 95% point‐wise CIs for the melanoma recurrence sub‐population. For this group of individuals, their baseline survival function decreases faster over the first five years, and then more slowly for the remainder of the follow‐up period.

The ability to make predictions based on an estimated mixture cure survival function is a key strength of the method presented in this article. In fact, values of the mixture cure survival function can be easily computed from our regression parameter estimates (both incidence and latency) and the estimate for the conditional baseline hazard function. For example, the estimated probability of a person with a Breslow thickness ≤0.8 mm (all other covariates are fixed at their mean values) having no recurrence for 5 years is 0.95 (95% CI: 0.93, 0.96), while this probability for a person with a Breslow thickness >0.8 mm is down to 0.91 (95% CI: 0.86, 0.94). Furthermore, the probabilities of a patient having no recurrence for 10 years are 0.93 (95% CI: 0.91, 0.94) and 0.88 (95% CI: 0.82, 0.93), respectively, for Breslow thickness ≤0.8 mm and >0.8 mm.

**FIGURE 3:**
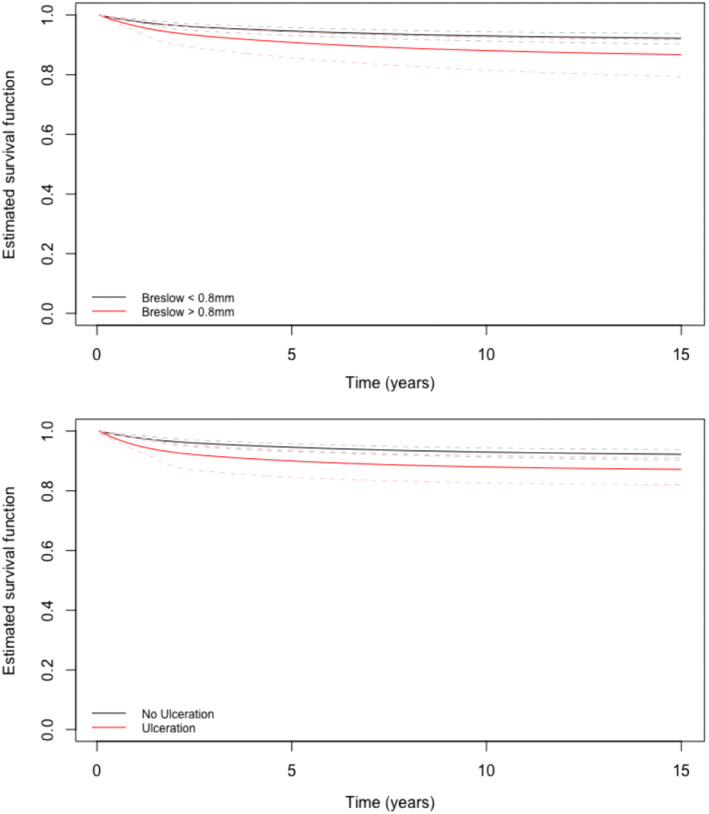
Estimated mixture survival functions for Breslow thickness ≤0.8 mm vs >0.8 mm and ulceration vs no ulceration, with point‐wise 95% confidence intervals

Similarly, based on the fitted mixture cure model, we can also easily compute entire predictive survival functions, and their point‐wise CIs. For example, Figure 3 displays mixture cure predictive survival functions illustrating the effect of Breslow thickness (top panel) and ulceration (bottom panel) with 95% point‐wise CIs. These plots explain that, at the population level, those with a Breslow thickness ≤0.8 mm are more likely to be free of recurrence at any time t than those with a Breslow thickness >0.8 mm, when all other covariates are set to the sample mean values. Similarly at the population level, those with no ulceration are more likely to be free of recurrence at any time t than those with ulceration. However, the survival differences between these groups may not be significantly different, as the associated point‐wise 95% CIs slightly overlap.

**FIGURE 4:**
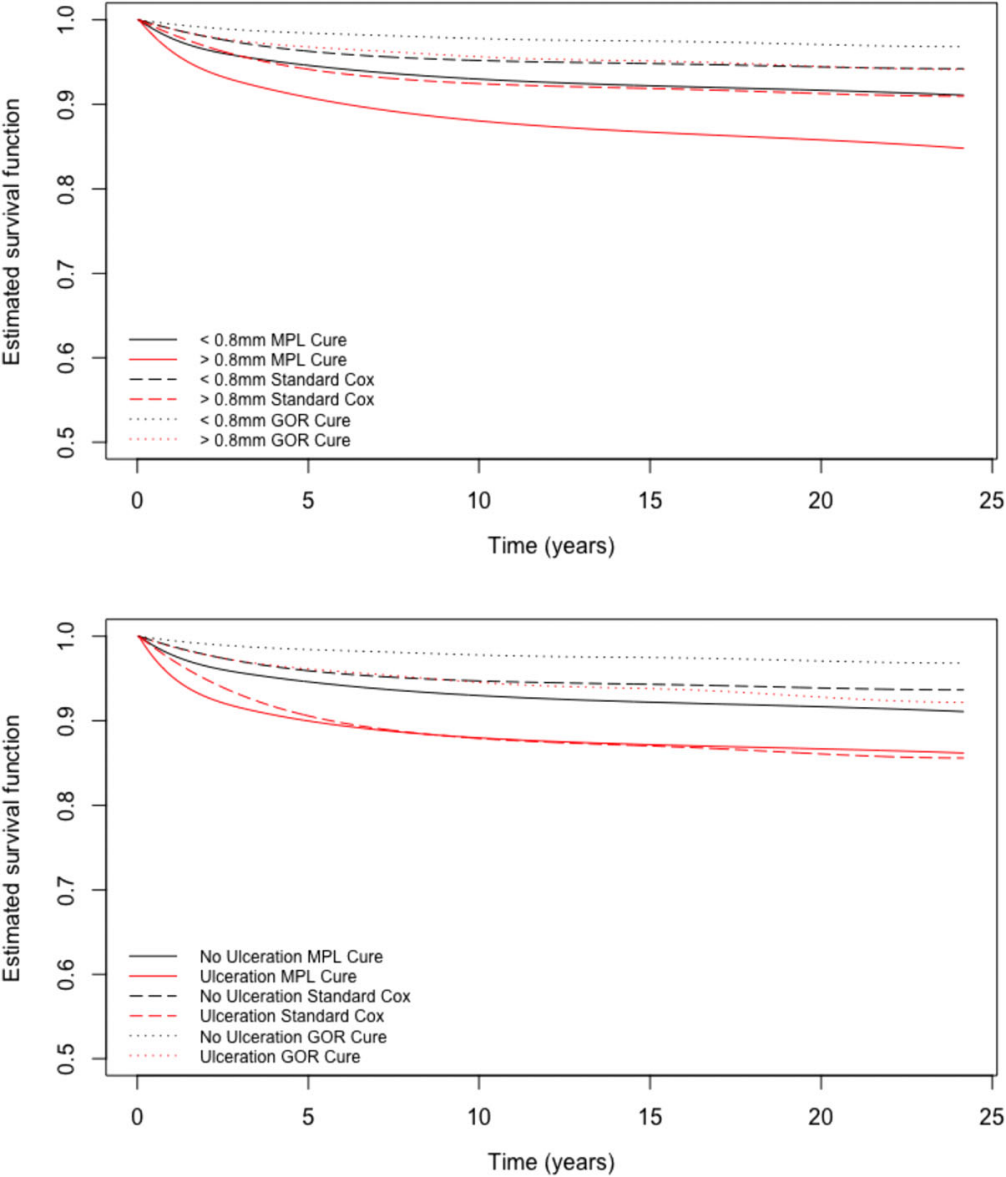
Estimated survival functions for Breslow thickness ≤0.8 mm vs >0.8 mm and ulceration vs no ulceration using the MPL Cox mixture cure model, GOR Cox mixture cure model, and a standard Cox model

We calculated again the predicted survival curves using GOR model from GORCure as well as the standard Cox model (so there was no cured fraction). The mixture cure survival functions from the MPL and GOR models and the standard Cox survival curves are displayed in Figure 4, comparing again the Breslow thickness groups (top panel) and the ulceration groups (bottom panel). The estimated survival functions from the GOR model are noticeably higher than their MPL counterparts. As seen in Section 5, the GOR model produces consistently negative bias in the estimation of the intercept of the incidence logistic regression model, which is equivalent to over‐estimating the size of the cured fraction and underestimating the risk of the event in the whole population. As a result, it appears that the survival function estimates produced by the GOR mixture cure model are too high. There are also clear discrepancies between the Cox and mixture cure predictive survival functions. Clearly, failing to account for the presence of a cured fraction also leads to an overestimation of survival probabilities in this thin melanoma example.

## 7 DISCUSSION AND CONCLUDING REMARKS

In this article, we propose a new penalized likelihood estimation method for fitting a mixture cure Cox model where observations are assumed partly interval‐censored. Our approach finds the MPL estimation of regression parameters for the latency and incidence models as well as estimation of the latency baseline hazard function which is approximated using M‐spline basis functions. The baseline hazard estimate is constrained to be non‐negative. One advantage of our method, when compared with the existing EM methods, is that it also yields an asymptotic covariance matrix for all the parameters, and hence allowing inference on both regression parameters and survival quantities. Another advantage of our method is that it produces smooth baseline hazard estimates. The results of simulation studies indicate that this method produces regression parameter and baseline hazard function estimates that perform well in terms of bias, variance and coverage probability. Our method avoids computationally intensive methods like bootstrapping which is adopted by the competitor EM‐algorithm for computing variances of the estimates. A package to implement the proposed method is available on Github, and we intend to upload the package to R CRAN in the near future.

Existing methods for checking the diagnostics of mixture cure models are sparse, particularly for cases involving partly interval censored data. Schoenfeld residuals^30^ are inappropriate for mixture cure models as the marginal hazards of any mixture cure model will be nonproportional. Wileyto et al^31^ developed pseudo‐residuals modeled on Schoenfeld to assess the fit of the latency model in the non‐cured fraction, but considered parametric mixture cure models for right censored data only. Peng and Taylor^32^ developed a modified martingale residual and a modified Cox‐Snell residual appropriate for checking the latency model of a mixture cure model, but again only considered right censored samples. Scolas et al^33^ developed a Cox‐Snell residual applicable to a parametric mixture cure model for interval censored data, which can be used to check both the uncured and mixture population survival distributions. These authors also developed a deviance residual appropriate for checking the linearity of both the incidence and latency parts of the model. However, the methods developed by Scolas et al^33^ are only appropriate for cases where the data is entirely right‐ or interval‐censored. Evidently, more work is required to derive appropriate residuals for diagnostic checks of the mixture cure Cox model proposed here.

We plan to extend the approach discussed in the article to other mixture cure survival models, particularly mixture cure additive hazards (AH) models and mixture cure accelerated failure time (AFT) models, where partly interval censoring times will be adopted. MPL estimations of AH and AFT models from partly interval censored survival times have been explored by Li and Ma,^34^, ^35^ and these computational algorithms will be extended to their mixture cure counterparts.

## Supporting information


**Data S1** Supplementary Materials

## Data Availability

The code used for the simulation studies and the application to the thin melanoma dataset is available publicly at https://github.com/annabelwebb/phmc_mpl/. The thin melanoma dataset may be accessed upon request from the Melanoma Institute Australia.
