# Supplementary material for: Penalized likelihood estimation of a mixture cure Cox model with partly interval censoring—An application to thin melanoma
Source: Stat Med. 2022 Apr 26;41(17):3260–80. doi: 10.1002/sim.9415 (PMC9544451; doi:10.1002/sim.9415)
Supplement: Supplementary file 1 — Data S1 Supplementary Materials [file SIM-41-3260-s001.pdf]

Supplementary Material for "Penalised likelihood estimation of a mixture cure Cox model with partly-interval censoring - an application to thin melanoma"

Annabel Webb[1], Jun Ma *[2], and Serigne Lǒ[3,4]

[1,2]Department of Mathematics and Statistics, Macquarie University, Sydney, Australia

[3]Melanoma Institute Australia, The University of Sydney, North Sydney, NSW, Australia

[4]Faculty of Medicine and Health, The University of Sydney, Sydney, NSW, Australia

1 Score vector and Hessian matrix elements

The components of the score vector of the penalised likelihood are as follows. Let z_{ik} be element k of vector $\mathbf{z}_i$ and let x_{ij} be element j of vector $\mathbf{x}_i$, for $k = 1, ..., p$ and $j = 1, ..., q$. Then the first derivative of $\Phi(\boldsymbol{\eta})$ with respect to β_j is

$$\frac{\partial \Phi(\boldsymbol{\eta})}{\partial \beta_j} = \sum_{i=1}^{n} z_{ij} \left((1 - \delta_i^R)(1 - \pi(\mathbf{z}_i)) + \delta_i^R \frac{(S(t) - 1)\pi(\mathbf{z}_i)(1 - \pi(\mathbf{z}_i))}{1 - \pi(\mathbf{z}_i) + \pi(\mathbf{z}_i)S(t)} \right) \tag{1}$$

The first derivative of $\Phi(\boldsymbol{\eta})$ with respect to γ_k is

$$\begin{aligned} \frac{\partial \Phi(\boldsymbol{\eta})}{\partial \gamma_k} = \sum_{i=1}^{n} x_{ik} \Bigg(& \delta_i(1 - H(t) - \delta_i^R \frac{\pi(\mathbf{z}_i)S(t)H(t)}{1 - \pi(\mathbf{z}_i) + \pi(\mathbf{z}_i)S(t)} \\ & + \delta_i^L \frac{S(t)H(t)}{1 - S(t)} - \delta_i^I \frac{S(t^L)H(t^L) - S(t^R)H(t^R)}{S(t^L) - S(t^R)} \Bigg) \end{aligned} \tag{2}$$

The first derivative of $\Phi(\boldsymbol{\eta})$ with respect to θ_u is

$$\begin{aligned} \frac{\partial \Phi(\boldsymbol{\eta})}{\partial \theta_u} = \sum_{i=1}^{n} \delta_i \frac{\psi_u(t)}{h_0(t)} - e^{x^\top \gamma} \Bigg(& \delta_i \Psi_u(t) + \delta_i^R \frac{\pi(\mathbf{z}_i)S(t)\Psi_u(t)}{1 - \pi(\mathbf{z}_i) + \pi(\mathbf{z}_i)S(t)} \\ & - \delta_i^L \frac{S(t)\Psi_u(t)}{1 - S(t)} + \delta_i^I \frac{S(t^L)\Psi_u(t^L) - S(t^R)\Psi_u(t^R)}{S(t^L) - S(t^R)} \Bigg) \end{aligned} \tag{3}$$

*To whom correspondence should be addressed: jun.ma@mq.edu.au

The components on the Hessian matrix are as follows.

$$\frac{\partial^2\Phi(\boldsymbol{\eta})}{\partial\beta_j\partial\beta_t} = -\sum_{i=1}^{n} z_{ij}z_{it}\Bigg((1-\delta_i^R)\pi(\mathbf{z}_i)(1-\pi(\mathbf{z}_i)) + \delta_i^R\frac{(S(t)-1)\pi(\mathbf{z}_i)(1-\pi(\mathbf{z}_i))^3}{(1-\pi(\mathbf{z}_i)+\pi(\mathbf{z}_i)S(t))^2}\Bigg) \tag{4}$$

$$\frac{\partial^2\Phi(\boldsymbol{\eta})}{\partial\beta_j\partial\gamma_k} = -\sum_{i=1}^{n} z_{ij}x_{ik}\delta_i^R\frac{S(t)H(t)\pi(\mathbf{z}_i)(1-\pi(\mathbf{z}_i))}{(1-\pi(\mathbf{z}_i)+\pi(\mathbf{z}_i)S(t))^2} \tag{5}$$

$$\frac{\partial^2\Phi(\boldsymbol{\eta})}{\partial\beta_j\partial\theta_u} = -\sum_{i=1}^{n} e^{x^\top\gamma}z_{ij}\delta_i^R\frac{S(t)\pi(\mathbf{z}_i)(1-\pi(\mathbf{z}_i))\Psi_u(t)}{(1-\pi(\mathbf{z}_i)+\pi(\mathbf{z}_i)S(t))^2} \tag{6}$$

$$\begin{aligned}\frac{\partial^2\Phi(\boldsymbol{\eta})}{\partial\gamma_k\partial\gamma_s} = -\sum_{i=1}^{n} x_{ik}x_{is}\Bigg(&\delta_i H(t) + \delta_i^R\frac{\pi(\mathbf{z}_i)S(t)H(t)}{1-\pi(\mathbf{z}_i)+\pi(\mathbf{z}_i)S(t)} \\ &-\delta_i^R\frac{\pi(\mathbf{z}_i)(1-\pi(\mathbf{z}_i))S(t)H^2(t)}{(1-\pi(\mathbf{z}_i)+\pi(\mathbf{z}_i)S(t))^2} - \delta_i^L\frac{S(t)H(t)}{1-S(t)} + \delta_i^L\frac{S(t)H^2(t)}{(1-S(t))^2} \\ &+\delta_i^I\frac{S(t^L)H(t^L)-S(t^R)H(t^R)}{S(t^L)-S(t^R)} + \delta_i^I\frac{S(t^L)S(t^R)(H(t^L)-H(t^R))^2}{(S(t^L)-S(t^R))^2}\Bigg)\end{aligned} \tag{7}$$

$$\begin{aligned}\frac{\partial^2\Phi(\boldsymbol{\eta})}{\partial\gamma_k\partial\theta_u} = -\sum_{i=1}^{n} x_{ij}e^{x^T\gamma}\Bigg(&\delta_I\Psi_u(t) + \delta_i^R\frac{\pi(\mathbf{z}_i)S(t)\Psi_u(t)}{1-\pi(\mathbf{z}_i)+\pi(\mathbf{z}_i)S(t)} \\ &-\delta_i^R\frac{\pi(\mathbf{z}_i)(1-\pi(\mathbf{z}_i))S(t)H(t)\Psi_u(t)}{(1-\pi(\mathbf{z}_i)+\pi(\mathbf{z}_i)S(t))^2} - \delta_i^L\frac{S(t)\Psi_u(t)}{1-S(t)} \\ &+\delta_i^L\frac{S(t)H(t)\Psi_u(t)}{(1-S(t))^2} + \delta_i^I\frac{S(t^L)\Psi_u(t^L)-S(t^R)\Psi_u(t^R)}{S(t^L)-S(t^R)} \\ &+\delta_i^I\frac{S(t^L)S(t^R)(H(t^L)-H(t^R))(\Psi_u(t^L)-\Psi_u(t^R))}{(S(t^L)-S(t^R))^2}\Bigg)\end{aligned} \tag{8}$$

$$\begin{aligned}\frac{\partial^2\Phi(\boldsymbol{\eta})}{\partial\theta_u\partial\theta_z} = -\sum_{i=1}^{n}\delta_i\frac{\psi_u(t)\psi_z(t)}{h_0^2(t)} - e^{x^T\gamma}\Bigg(&\delta_i^R\frac{\pi(\mathbf{z}_i)(1-\pi(\mathbf{z}_i))S(t)\Psi_u(t)\Psi_z(t)}{(1-\pi(\mathbf{z}_i)+\pi(\mathbf{z}_i)S(t))^2} \\ -\delta_i^L\frac{S(t)\Psi_u(t)\Psi_z(t)}{(1-S(t))^2} - \delta_i^I&\frac{S(t^L)S(t^R)(\Psi_u(t^L)-\Psi_u(t^R))(\Psi_z(t^L)-\Psi_z(t^R))}{(S(t^L-S(t^R))^2}\Bigg)\end{aligned} \tag{9}$$

2 Proof for Theorem 1

Let a and b be the minimum and maximum of all the observed survival times respectively, including interval censoring but excluding 0 and ∞. Then, let $C^r[a,b]$ be the set of functions that have r continuous derivatives over $[a,b]$. The parameter space for $\boldsymbol{\beta}$ can be given by $B = \{\boldsymbol{\beta} : |\beta_t| \leq C_1 < \infty, \forall t\}$. The parameter space for $\boldsymbol{\gamma}$ can be given by $G = \{\boldsymbol{\gamma} : |\gamma_j| \leq C_2 < \infty, \forall j\}$. The parameter space for $h_0(t)$ can be given by $A = \{h_0(t) : h_0 \in C^r[a,b], 0 \leq h_0(t)] \leq C_3 < \infty, \forall t \in [a,b]\}$. Therefore, the parameter space for $\boldsymbol{\tau} = (\boldsymbol{\beta}, \boldsymbol{\gamma}, h_0(t))$ is $\boldsymbol{\Gamma} = \{\boldsymbol{\tau} : \boldsymbol{\beta} \in B, \boldsymbol{\gamma} \in G, h_0 \in A\}$. Before defining the MPL estimator of $\boldsymbol{\tau}$, it is necessary to account for the fact that this method estimates an approximation of $h_0(t)$. For convenience, the approximation can be denoted as $\tilde{h}_0(t) = \sum_{u=1}^{m}\theta_u\psi_u(t)$. The parameter space for $\tilde{h}_0(t)$ can be given by $A_n = \{\tilde{h}_0(t) : 0 \leq \tilde{h}_0(t) \leq C_4 < \infty, \forall t \in [a,b]\}$. Then the parameter space for $\boldsymbol{\tau}_n$ is $\boldsymbol{\Gamma}_n = \{\boldsymbol{\tau}_n : \boldsymbol{\beta} \in B, \boldsymbol{\gamma} \in G, \tilde{h}_0 \in A_n\}$. The MPL estimator of $\boldsymbol{\tau}_n$ is then $\hat{\boldsymbol{\tau}}_n = (\hat{\boldsymbol{\beta}}, \hat{\boldsymbol{\gamma}}, \hat{h}_0(t))$. Theorem 1 demonstrates asymptotic consistency for $\hat{\boldsymbol{\tau}}_n$ when the number of basis functions

$m \to \infty$ but $m/n \to 0$ when $n \to \infty$, and the scaled smoothing value $\mu_n = \lambda/n \to 0$ when $n \to \infty$.

Theorem 1 requires the following regularity conditions:

B1. The matrices $\mathbf{X}$ and $\mathbf{Z}$ are bounded, and both $E(\mathbf{X}\mathbf{X}^T)$ and $E(\mathbf{Z}\mathbf{Z}^T)$ are non-singular.

B2. The penalty function $J(\boldsymbol{\eta})$ is bounded over $\boldsymbol{\Gamma}$ and $\boldsymbol{\Gamma}_n$.

B3. For function $\tilde{h}_0(t)$, there is a constant C_5 independent of n that is the upper bound of all $\theta_u \geq 0$. Additionally, the basis functions $\psi_u(t)$, where $u = 1, ..., m$, are bounded for $t \in [a, b]$.

B4. The knots and basis functions are selected such that for any $h_0(t) \in A$ there is a $\tilde{h}_0(t) \in A_n$ which satisfies $\max_t |\tilde{h}_0(t) - h_0(t)| \to 0$ when $n \to \infty$.

Define the distance measure $\rho(\boldsymbol{\tau}_1, \boldsymbol{\tau}_2)$ as

$$\rho(\boldsymbol{\tau}_1, \boldsymbol{\tau}_2) = \{\|\boldsymbol{\tau}_1 - \boldsymbol{\tau}_1\|^2\}^{1/2} = \left\{ \|\boldsymbol{\beta}_1 - \boldsymbol{\beta}_2\|_2^2 + \|\boldsymbol{\gamma}_1 - \boldsymbol{\gamma}_2\|_2^2 + \sup_{t \in [a,b]} |h_{01}(t) - h_{02}(t)|^2 \right\}^{1/2}$$

Under the above regularity conditions, Theorem 1 can be demonstrated by showing that $\rho(\boldsymbol{\tau}_0, \hat{\boldsymbol{\tau}}_n) \to 0$ almost surely, where $\boldsymbol{\tau}_0 = (\boldsymbol{\beta}_0, \boldsymbol{\gamma}_0, h_{00}(t))$ is the true parameter value. The required result can be obtained by applying Theorem 1 from (1).

3 Proof for Theorem 2

Theorem 2 can be shown under the following regularity conditions:

1. The distributions of $\mathbf{x}_i$ and $\mathbf{z}_i$ are independent of $\boldsymbol{\eta}$.

2. The limit $\lim_{n\to\infty}[n^{-1}l(\boldsymbol{\eta})]$ exists and has a unique maximum at $\boldsymbol{\eta}_0 \in \Omega$, where Ω is the parameter space for $\boldsymbol{\eta}$ and is a compact subspace of $\mathbf{R}^{p+q+m}$. That is to say, if the sample size is infinity, the true parameters can be obtained exactly from maximising the likelihood.

3. $l(\boldsymbol{\eta})$ has a finite upper bound and is twice continuously differentiable in a neighbourhood of $\boldsymbol{\eta}_0$, and the matrices

$$\lim_{n\to\infty} n^{-1} \sum_{i=1}^{n} \frac{\partial l_i(\boldsymbol{\eta})}{\partial \boldsymbol{\eta}} \frac{\partial l_i(\boldsymbol{\eta})}{\partial \boldsymbol{\eta}^T}$$

   and

$$\lim_{n\to\infty} \left[-n^{-1} \frac{\partial^2(\boldsymbol{\eta})}{\partial \boldsymbol{\eta} \partial \boldsymbol{\eta}^T} \right]$$

   exist.

4. The penalty function $J(\boldsymbol{\eta})$ is twice continuously differentiable on Ω, and these derivatives are bounded.

5. The matrix $\mathbf{U}^T\mathbf{F}(\boldsymbol{\eta})\mathbf{U}$ is invertible in a neighbourhood of $\boldsymbol{\eta}_0$.

If these conditions hold, it is a simple matter to demonstrate that $\hat{\boldsymbol{\eta}} \to \boldsymbol{\eta}_0$. Let $\bar{l}(\boldsymbol{\eta}) = \lim_{n\to\infty}[n^{-1}l(\boldsymbol{\eta})]$ exist with a unique maximum at $\boldsymbol{\eta}_0 \in \Omega$, where Ω is the parameter space for $\boldsymbol{\eta}$. Under the strong law of large numbers, we have $n^{-1}l(\boldsymbol{\eta}) \to \bar{l}(\boldsymbol{\eta})$ almost surely and uniformly for $\boldsymbol{\eta} \in \Omega$. Additionally, we have $\mu_n \to 0$ as $n \to \infty$. This is sufficient to show that $\hat{\boldsymbol{\eta}} \to \boldsymbol{\eta}_0$.

The asymptotic normality result can be proven by following Theorem 2 from (1). Given that, according to the KKT conditions outlined above in Section 3.4, we have a constrained MPL estimate $\hat{\boldsymbol{\eta}}$ that satisfies

$$\mathbf{U}^T \frac{\partial \Phi(\hat{\boldsymbol{\eta}})}{\partial \boldsymbol{\eta}} = 0$$

it is possible to show that

$$\sqrt{n}(\hat{\boldsymbol{\eta}} - \boldsymbol{\eta}_0) = -\mathbf{U}\left(\mathbf{U}^T \frac{1}{n}\frac{\partial^2 \Phi(\tilde{\boldsymbol{\eta}})}{\partial \boldsymbol{\eta}\partial \boldsymbol{\eta}^T}\mathbf{U}\right)^{-1} \mathbf{U}^T \left(\frac{1}{\sqrt{n}}\frac{\partial l(\boldsymbol{\eta}_0)}{\partial \boldsymbol{\eta}} + o(1)\right)$$

where $\tilde{\boldsymbol{\eta}}$ is a vector between $\hat{\boldsymbol{\eta}}$ and $\boldsymbol{\eta}_0$. Here, when $n \to \infty$ and $\mu_n \to 0$, $n^{-1}\partial^2\Phi(\tilde{\boldsymbol{\eta}})/\partial\boldsymbol{\eta}\partial\boldsymbol{\eta}^T$ converges almost surely to $F(\boldsymbol{\eta}_0)$ under the law of large numbers. If we then apply the central limit theorem to $n^{-1/2}\partial l(\boldsymbol{\eta}_0)/\partial\boldsymbol{\eta}$, the asymptotic normality result is demonstrated.

4 Additional simulation study results

Table 1: Study 1 (partly-interval censoring): Cox and logistic regression parameters for $n = 200$ and $n = 1000$ for Scenario 2 (Exponential baseline).

			$1-\pi(\mathbf{z})=0.2$									$1-\pi(\mathbf{z})=0.6$								
			$\pi^E=0.65$			$\pi^E=0.38$			$\pi^E=0.0$			$\pi^E=0.65$			$\pi^E=0.38$			$\pi^E=0.0$		
			Bias	SE	CP	Bias	SE	CP	Bias	SE	CP	Bias	SE	CP	Bias	SE	CP	Bias	SE	CP
$n=200$	β_0	MPL	0.058	0.293	0.95	0.079	0.302	0.96	0.080	0.308	0.95	0.015	0.225	0.96	-0.004	0.257	0.96	0.019	0.52	0.97
			(0.039)	(0.300)		(0.053)	(0.301)		(0.053)	(0.310)		(0.031)	(0.198)		(0.007)	(0.194)		(0.038)	(0.199)	
		GOR	-0.498	0.104	0.01	-0.472	0.238	0.47	-0.468	0.293	0.59	-0.483	0.233	0.49	-0.519	0.235	0.36	-0.603	0.217	0.28
			(0.332)	(0.111)		(0.314)	(0.245)		(0.312)	(0.329)		(0.966)	(0.250)		(1.038)	(0.222)		(1.207)	(0.265)	
	β_1	MPL	0.060	0.495	0.97	0.054	0.505	0.96	0.076	0.507	0.96	0.019	0.355	0.96	0.034	0.368	0.97	0.018	0.362	0.96
			(0.060)	(0.467)		(0.054)	(0.525)		(0.076)	(0.522)		(0.019)	(0.352)		(0.034)	(0.337)		(0.018)	(0.356)	
		GOR	0.006	0.172	0.96	0.028	0.396	0.96	0.065	0.491	0.96	-0.010	0.314	0.94	0.054	0.316	0.94	0.038	0.287	0.82
			(0.006)	(0.174)		(0.028)	(0.396)		(0.065)	(0.521)		(0.010)	(0.335)		(0.054)	(0.331)		(0.038)	(0.340)	
	β_2	MPL	-0.024	0.239	0.94	-0.044	0.243	0.95	-0.021	0.244	0.95	-0.013	0.189	0.94	-0.014	0.194	0.97	-0.012	0.195	0.97
			(0.048)	(0.251)		(0.089)	(0.253)		(0.042)	(0.251)		(0.026)	(0.193)		(0.029)	(0.176)		(0.024)	(0.182)	
		GOR	0.016	0.086	0.93	-0.002	0.197	0.94	-0.016	0.237	0.95	0.001	0.165	0.97	-0.018	0.166	0.95	-0.018	0.151	0.84
			(0.032)	(0.092)		(0.004)	(0.196)		(0.032)	(0.249)		(0.001)	(0.169)		(0.036)	(0.174)		(0.037)	(0.169)	
	γ_1	MPL	0.031	0.203	0.94	0.11	0.205	0.95	0.016	0.206	0.96	0.046	0.358	0.93	0.049	0.402	0.94	0.042	0.388	0.95
			(0.063)	(0.208)		(0.023)	(0.209)		(0.033)	(0.206)		(0.092)	(0.334)		(0.097)	(0.402)		(0.083)	(0.327)	
		GOR	0.007	0.074	0.95	0.029	0.169	0.96	-0.001	0.199	0.94	0.001	0.247	0.94	0.011	0.255	0.96	-0.362	0.259	0.80
			(0.015)	(0.077)		(0.059)	(0.168)		(0.002)	(0.210)		(0.002)	(0.251)		(0.022)	(0.266)		(0.725)	(0.284)	
	γ_2	MPL	0.055	0.125	0.91	0.069	0.128	0.93	0.056	0.126	0.92	0.098	0.250	0.94	0.095	0.300	0.93	0.088	0.301	0.94
			(0.055)	(0.135)		(0.069)	(0.130)		(0.056)	(0.134)		(0.098)	(0.220)		(0.095)	(0.218)		(0.088)	(0.208)	
		GOR	0.012	0.048	0.96	0.016	0.109	0.96	0.023	0.125	0.92	0.011	0.158	0.98	0.006	0.164	0.94	-0.711	0.182	0.26
			(0.012)	(0.045)		(0.016)	(0.106)		(0.023)	(0.173)		(0.011)	(0.184)		(0.006)	(0.210)		(0.711)	(0.489)	
$n=1000$	β_0	MPL	0.008	0.120	0.96	0.027	0.122	0.93	0.023	0.121	0.96	0.006	0.091	0.96	0.003	0.089	0.95	-0.003	0.089	0.95
			(0.006)	(0.121)		(0.018)	(0.127)		(0.015)	(0.119)		(0.012)	(0.082)		(0.006)	(0.086)		(0.005)	(0.086)	
		GOR	-0.491	0.104	0.01	-0.503	0.104	0.01	-0.484	0.128	0.06	-0.494	0.103	0.00	-0.498	0.103	0.00	-0.532	0.112	0.01
			(0.327)	(0.106)		(0.336)	(0.107)		(0.323)	(0.132)		(0.988)	(0.106)		(0.996)	(0.106)		(1.063)	(0.132)	
	β_1	MPL	0.002	0.204	0.95	0.033	0.207	0.95	0.006	0.205	0.97	0.018	0.153	0.92	0.002	0.153	0.96	-0.005	0.153	0.95
			(0.002)	(0.209)		(0.033)	(0.212)		(0.006)	(0.202)		(0.018)	(0.162)		(0.002)	(0.155)		(0.005)	(0.153)	
		GOR	0.003	0.172	0.95	0.026	0.172	0.93	0.004	0.205	0.97	-0.008	0.139	0.95	0.005	0.139	0.96	-0.019	0.147	0.91
			(0.003)	(0.181)		(0.026)	(0.180)		(0.004)	(0.202)		(0.008)	(0.141)		(0.005)	(0.130)		(0.019)	(0.153)	
	β_2	MPL	-0.001	0.100	0.94	-0.012	0.102	0.95	-0.011	0.102	0.97	0.001	0.080	0.95	-0.006	0.080	0.96	-0.005	0.080	0.95
			(0.001)	(0.104)		(0.025)	(0.106)		(0.023)	(0.100)		(0.003)	(0.076)		(0.011)	(0.078)		(0.010)	(0.083)	
		GOR	-0.007	0.086	0.93	0.001	0.086	0.97	-0.011	0.101	0.97	0.005	0.072	0.96	0.003	0.072	0.97	0.004	0.077	0.92
			(0.014)	(0.088)		(0.001)	(0.082)		(0.021)	(0.100)		(0.010)	(0.069)		(0.005)	(0.072)		(0.007)	(0.083)	
	γ_1	MPL	0.008	0.088	0.96	0.007	0.088	0.96	0.007	0.088	0.94	0.009	0.136	0.95	0.021	0.136	0.93	0.010	0.136	0.95
			(0.015)	(0.090)		(0.015)	(0.088)		(0.014)	(0.091)		(0.018)	(0.138)		(0.041)	(0.142)		(0.020)	(0.133)	
		GOR	-0.002	0.074	0.97	0.002	0.076	0.97	0.005	0.087	0.93	0.003	0.107	0.98	-0.020	0.108	0.94	-0.096	0.127	0.75
			(0.003)	(0.072)		(0.004)	(0.075)		(0.009)	(0.090)		(0.006)	(0.099)		(0.040)	(0.110)		(0.193)	(0.234)	
	γ_2	MPL	0.015	0.055	0.93	0.018	0.055	0.95	0.018	0.055	0.94	0.029	0.089	0.93	0.031	0.089	0.93	0.022	0.088	0.96
			(0.015)	(0.056)		(0.018)	(0.052)		(0.018)	(0.054)		(0.029)	(0.864)		(0.031)	(0.081)		(0.022)	(0.083)	
		GOR	0.010	0.048	0.95	0.001	0.050	0.95	0.013	0.054	0.94	0.002	0.067	0.99	-0.002	0.069	0.95	-0.185	0.081	0.76
			(0.010)	(0.045)		(0.00)	(0.053)		(0.013)	(0.054)		(0.002)	(0.058)		(0.002)	(-)		(0.185)	(0.412)	

SE = standard error, CP = coverage probability

Table 2: Study 1 (partly-interval censoring): baseline survival function estimation for $n = 200$ and $n = 1000$ for Scenario 2 (exponential baseline).

			$1-\pi(\mathbf{z})=0.2$									$1-\pi(\mathbf{z})=0.6$								
			$\pi^E=0.65$			$\pi^E=0.38$			$\pi^E=0.0$			$\pi^E=0.65$			$\pi^E=0.38$			$\pi^E=0.0$		
			Bias	SE	CP	Bias	SE	CP	Bias	SE	CP	Bias	SE	CP	Bias	SE	CP	Bias	SE	CP
$n=200$	t_1	MPL	0.002	0.041	0.94	0.004	0.042	0.94	0.001	0.041	0.94	0.012	0.072	0.94	0.010	0.085	0.91	0.006	0.077	0.92
				(0.046)	(0.97)		(0.045)	(0.95)		(0.043)	(0.96)		(0.072)	(0.97)		(0.089)	(1.00)		(0.072)	(0.96)
		GOR	-0.317	-	-	-0.434	-	-	0.097	-	-	-0.477	-	-	-0.481	-	-	0.011	-	-
				(0.106)	(0.16)		(0.111)	(0.04)		(0.069)	(0.70)		(0.104)	(0.02)		(0.110)	(0.04)		(0.095)	(0.94)
	t_2	MPL	-0.001	0.030	0.91	-0.003	0.031	0.90	0.003	0.033	0.92	0.003	0.063	0.88	0.001	0.072	0.88	0.006	0.073	0.90
				(0.033)	(0.96)		(0.034)	(0.95)		(0.034)	(0.95)		(0.058)	(0.96)		(0.057)	(0.96)		(0.059)	(0.95)
		GOR	0.047	-	-	-0.060	-	-	0.069	-	-	-0.130	-	-	-0.142	-	-	0.025	-	-
				(0.042)	(0.79)		(0.126)	(0.90)		(0.059)	(0.79)		(0.152)	(0.85)		(0.149)	(0.81)		(0.070)	(0.92)
	t_3	MPL	0.002	0.019	0.82	-0.001	0.020	0.86	0.002	0.023	0.87	0.008	0.048	0.78	0.006	0.058	0.78	0.008	0.054	0.78
				(0.022)	(0.95)		(0.021)	(0.95)		(0.021)	(0.95)		(0.042)	(0.94)		(0.043)	(0.94)		(0.043)	(0.94)
		GOR	0.026	-	-	0.024	-	-	0.040	-	-	0.008	-	-	0.009	-	-	0.012	-	-
				(0.001)	(0.00)		(0.015)	(0.60)		(0.043)	(0.86)		(0.040)	(0.94)		(0.040)	(0.94)		(0.055)	(0.93)
$n=1000$	t_1	MPL	0.001	0.019	0.94	0.001	0.019	0.95	0.001	0.018	0.96	0.003	0.029	0.95	0.003	0.030	0.95	0.004	0.029	0.96
				(0.021)	(0.96)		(0.019)	(0.96)		(0.020)	(0.97)		(0.032)	(0.97)		(0.031)	(0.97)		(0.029)	(0.97)
		GOR	-0.336	-	-	-0.340	-	-	0.101	-	-	-0.382	-	-	-0.383	-	-	-0.008	-	-
				(0.112)	(0.14)		(0.111)	(0.16)		(0.028)	(0.05)		(0.108)	(0.07)		(0.117)	(0.13)		(0.528)	(0.98)
	t_2	MPL	-0.001	0.013	0.94	0.001	0.014	0.94	0.001	0.014	0.94	0.003	0.022	0.96	0.002	0.023	0.96	0.001	0.023	0.95
				(0.014)	(0.97)		(0.014)	(0.95)		(0.015)	(0.96)		(0.023)	(0.96)		(0.023)	(0.96)		(0.023)	(0.96)
		GOR	0.034	-	-	0.039	-	-	0.066	-	-	0.010	-	-	0.008	-	-	-0.022	-	-
				(0.042)	(0.96)		(0.050)	(0.99)		(0.025)	(0.20)		(0.059)	(0.97)		(0.073)	(0.96)		(0.491)	(0.98)
	t_3	MPL	0.001	0.008	0.92	0.001	0.009	0.93	0.001	0.009	0.91	0.002	0.015	0.91	0.002	0.017	0.93	0.001	0.016	0.92
				(0.008)	(0.96)		(0.009)	(0.96)		(0.009)	(0.96)		(0.015)	(0.94)		(0.016)	(0.95)		(0.016)	(0.96)
		GOR	0.020	-	-	0.026	-	-	0.035	-	-	0.022	-	-	0.027	-	-	-0.048	-	-
				(0.001)	(0.00)		(0.001)	(0.00)		(0.019)	(0.58)		(0.002)	(0.01)		(0.003)	(0.01)		(0.476)	(0.98)

SE = standard error, CP = coverage probability

Table 3: Study 1 (partly-interval censoring): Cox and logistic regression parameters for $n = 200$ and $n = 1000$ for Scenario 3 (Log-logistic baseline).

			$1-\pi(\mathbf{z}) = 0.2$									$1-\pi(\mathbf{z}) = 0.6$								
			$\pi^E = 0.65$			$\pi^E = 0.38$			$\pi^E = 0.0$			$\pi^E = 0.65$			$\pi^E = 0.38$			$\pi^E = 0.0$		
			Bias	SE	CP	Bias	SE	CP	Bias	SE	CP	Bias	SE	CP	Bias	SE	CP	Bias	SE	CP
$n = 200$	β_0	MPL	0.057	0.273	0.96	0.062	0.278	0.94	0.077	0.288	0.98	-0.013	0.208	0.95	-0.007	0.208	0.95	-0.017	0.209	0.96
			(0.038)	(0.288)		(0.042)	(0.299)		(0.051)	(0.297)		(0.026)	(0.187)		(0.014)	(0.183)		(0.033)	(0.191)	
		GOR	-0.455	0.262	0.50	-0.489	0.264	0.500	-0.516	0.265	0.52	-0.531	0.244	0.39	-0.525	0.249	0.47	-0.586	0.224	0.31
			(0.303)	(0.275)		(0.326)	(0.278)		(0.344)	(0.386)		(1.062)	(0.264)		(1.050)	(0.272)		(1.171)	(0.262)	
	β_1	MPL	0.057	0.473	0.97	0.051	0.473	0.96	0.062	0.487	0.97	0.024	0.347	0.94	0.001	0.346	0.95	0.017	0.348	0.94
			(0.057)	(0.496)		(0.051)	(0.472)		(0.062)	(0.501)		(0.024)	(0.340)		(0.001)	(0.349)		(0.017)	(0.347)	
		GOR	0.032	0.441	0.96	0.034	0.439	0.96	0.005	0.446	0.91	-0.003	0.330	0.93	0.020	0.332	0.94	0.018	0.301	0.85
			(0.032)	(0.420)		(0.034)	(0.480)		(0.005)	(0.541)		(0.003)	(0.357)		(0.020)	(0.354)		(0.018)	(0.353)	
	β_2	MPL	-0.025	0.224	0.95	-0.036	0.231	0.94	-0.038	0.234	0.96	-0.018	0.182	0.95	-0.011	0.184	0.95	-0.025	0.182	0.95
			(0.050)	(0.233)		(0.089)	(0.253)		(0.077)	(0.246)		(0.037)	(0.183)		(0.022)	(0.184)		(0.051)	(0.186)	
		GOR	-0.044	0.217	0.94	-0.014	0.219	0.98	-0.003	0.216	0.89	-0.026	0.173	0.94	-0.009	0.175	0.95	-0008	0.158	0.86
			(0.087)	(0.234)		(0.027)	(0.224)		(0.005)	(0.273)		(0.052)	(0.179)		(0.017)	(0.191)		(0.015)	(0.196)	
	γ_1	MPL	0.001	0.191	0.96	0.031	0.195	0.95	0.028	0.197	0.93	0.029	0.325	0.92	0.046	0.322	0.96	0.033	0.348	0.94
			(0.003)	(0.186)		(0.061)	(0.200)		(0.057)	(0.204)		(0.057)	(0.322)		(0.092)	(0.294)		(0.067)	(0.298)	
		GOR	0.020	0.186	0.95	-0.005	0.187	0.96	-0.011	0.181	0.87	0.013	0.276	0.94	0.019	0.293	0.96	-0.333	0.264	0.83
			(0.041)	(0.188)		(0.009)	(0.183)		(0.022)	(0.238)		(0.025)	(0.303)		(0.037)	(0.312)		(0.666)	(0.311)	
	γ_2	MPL	0.030	0.116	0.94	0.036	0.120	0.95	0.027	0.120	0.92	0.074	0.230	0.92	0.068	0.221	0.94	0.059	0.213	0.94
			(0.030)	(0.114)		(0.036)	(0.121)		(0.027)	(0.130)		(0.074)	(0.195)		(0.068)	(0.193)		(0.059)	(0.195)	
		GOR	0.020	0.118	0.94	0.026	0.122	0.94	0.045	0.113	0.87	-0.012	0.184	0.88	0.037	0.190	0.90	-0.691	0.185	0.28
			(0.020)	(0.128)		(0.026)	(0.125)		(0.045)	(0.282)		(0.012)	(0.313)		(0.037)	(0.290)		(0.691)	(0.498)	
$n = 1000$	β_0	MPL	0.004	0.113	0.93	0.016	0.116	0.97	0.005	0.115	0.93	0.005	0.090	0.96	-0.003	0.096	0.97	-0.004	0.096	0.96
			(0.002)	(0.120)		(0.011)	(0.115)		(0.004)	(0.121)		(0.010)	(0.079)		(0.003)	(0.080)		(0.009)	(0.081)	
		GOR	-0.513	0.117	0.01	-0.469	0.270	0.55	-0.564	0.115	0.04	-0.518	0.109	0.00	-0.529	0.110	0.01	-0.523	0.110	0.00
			(0.342)	(0.01)		(0.313)	(0.284)		(0.376)	(0.269)		(1.036)	(0.103)		(1.057)	(0.115)		(1.047)	(0.127)	
	β_1	MPL	-0.009	0.196	0.95	0.011	0.199	0.97	0.013	0.198	0.94	0.006	0.150	0.95	0.012	0.152	0.94	-0.004	0.151	0.96
			(0.009)	(0.200)		(0.011)	(0.195)		(0.013)	(0.197)		(0.006)	(0.150)		(0.012)	(0.157)		(0.004)	(0.148)	
		GOR	0.008	0.187	0.98	0.017	0.445	0.96	-0.048	0.186	0.89	-0.012	0.144	0.97	0.001	0.145	0.98	-0.015	0.146	0.94
			(0.008)	(0.174)		(0.017)	(0.436)		(0.048)	(0.311)		(0.012)	(0.136)		(0.001)	(0.132)		(0.015)	(0.150)	
	β_2	MPL	-0.011	0.097	0.96	-0.008	0.098	0.95	-0.013	0.098	0.94	-0.001	0.078	0.5	-0.005	0.079	0.94	-0.001	0.080	0.94
			(0.021)	(0.097)		(0.025)	(0.106)		(0.025)	(0.098)		(0.002)	(0.081)		(0.010)	(0.081)		(0.002)	(0.081)	
		GOR	0.001	0.093	0.96	-0.010	0.219	0.91	-0.020	0.092	0.89	-0.002	0.076	0.94	0.007	0.076	0.94	0.006	0.076	0.93
			(0.002)	(0.089)		(0.020)	(0.233)		(0.041)	(0.155)		(0.005)	(0.074)		(0.014)	(0.074)		(0.012)	(0.081)	
	γ_1	MPL	0.007	0.083	0.93	0.003	0.085	0.96	-0.004	0.085	0.94	0.009	0.129	0.95	-0.003	0.134	0.96	0.007	0.134	0.94
			(0.014)	(0.089)		(0.007)	(0.081)		(0.008)	(0.085)		(0.017)	(0.126)		(0.006)	(0.121)		(0.014)	(0.133)	
		GOR	0.009	0.082	0.92	0.014	0.189	0.94	0.037	0.080	0.88	0.011	0.119	0.92	0.019	0.121	0.92	-0.117	0.124	0.71
			(0.017)	(0.091)		(0.028)	(0.188)		(0.073)	(0.145)		(0.021)	(0.139)		(0.038)	(0.131)		(0.234)	(0.247)	
	γ_2	MPL	0.009	0.051	0.93	0.006	0.052	0.94	0.005	0.053	0.97	0.016	0.088	0.96	0.019	0.097	0.94	0.019	0.101	0.97
			(0.009)	(0.053)		(0.006)	(0.052)		(0.005)	(0.050)		(0.016)	(0.074)		(0.019)	(0.079)		(0.019)	(0.083)	
		GOR	0.008	0.052	0.96	0.023	0.121	0.95	-0.059	0.049	0.90	0.030	0.079	0.94	0.033	0.080	0.92	-0.230	0.081	0.69
			(0.008)	(0.051)		(0.023)	(0.117)		(0.059)	(0.247)		(0.030)	(0.085)		(0.033)	(0.085)		(0.230)	(0.444)	

SE = standard error, CP = coverage probability

Table 4: Study 1 (partly-interval censoring): baseline survival function estimation for $n = 200$ and $n = 1000$ for Scenario 2 (exponential baseline).

			$1-\pi(\mathbf{z})=0.2$									$1-\pi(\mathbf{z})=0.6$								
			$\pi^E=0.65$			$\pi^E=0.38$			$\pi^E=0.0$			$\pi^E=0.65$			$\pi^E=0.38$			$\pi^E=0.0$		
			Bias	SE	CP	Bias	SE	CP	Bias	SE	CP	Bias	SE	CP	Bias	SE	CP	Bias	SE	CP
$n=200$	t_1	MPL	0.005	0.036	0.91	0.009	0.037	0.93	0.007	0.037	0.93	0.006	0.067	0.91	0.009	0.063	0.92	0.011	0.063	0.93
				(0.067)	(1.00)		(0.063)	(1.00)		(0.062)	(1.00)		(0.095)	(1.00)		(0.097)	(1.00)		(0.092)	(0.99)
		GOR	0.036	-	-	0.079	-	-	0.105	-	-	0.031	-	-	0.028	-	-	0.033	-	-
				(0.062)	(0.84)		(0.059)	(0.62)		(0.081)	(0.79)		(0.097)	(0.91)		(0.100)	(0.89)		(0.104)	(0.93)
	t_2	MPL	-0.001	0.018	0.85	-0.003	0.022	0.89	0.002	0.022	0.86	0.004	0.040	0.82	-0.001	0.037	0.79	-0.001	0.041	0.78
				(0.023)	(0.97)		(0.025)	(0.98)		(0.026)	(0.98)		(0.043)	(0.96)		(0.044)	(0.97)		(0.043)	(0.97)
		GOR	-0.003	-	-	0.010	-	-	0.055	-	-	0.003	-	-	0.003	-	-	0.038	-	-
				(0.030)	(0.93)		(0.028)	(0.86)		(0.050)	(0.82)		(0.041)	(0.91)		(0.051)	(0.94)		(0.069)	(0.93)
	t_3	MPL	0.001	0.009	0.75	-0.001	0.011	0.78	0.001	0.012	0.76	0.004	0.023	0.68	0.004	0.021	0.67	0.003	0.027	0.63
				(0.010)	(0.93)		(0.012)	(0.96)		(0.013)	(0.94)		(0.023)	(0.95)		(0.025)	(0.96)		(0.023)	(0.96)
		GOR	-0.005	-	-	-0.001	-	-	0.027	-	-	-0.004	-	-	-0.006	-	-	0.016	-	-
				(0.016)	(0.91)		(0.015)	(0.94)		(0.032)	(0.88)		(0.022)	(0.94)		(0.037)	(0.98)		(0.059)	(0.95)
$n=1000$	t_1	MPL	0.003	0.015	0.93	0.003	0.016	0.94	0.003	0.017	0.95	0.007	0.026	0.93	0.003	0.028	0.95	0.003	0.028	0.94
				(0.034)	(1.00)		(0.032)	(1.00)		(0.030)	(1.00)		(0.046)	(1.00)		(0.045)	(1.00)		(0.046)	(1.00)
		GOR	0.011	-	-	0.032	-	-	0.100	-	-	0.031	-	-	0.015	-	-	0.079	-	-
				(0.026)	(0.80)		(0.059)	(0.88)		(0.036)	(0.14)		(0.041)	(0.78)		(0.044)	(0.86)		(0.068)	(0.85)
	t_2	MPL	-0.001	0.007	0.93	-0.001	0.009	0.93	0.001	0.009	0.92	0.001	0.013	0.91	-0.001	0.013	0.89	-0.001	0.014	0.92
				(0.009)	(0.99)		(0.011)	(0.98)		(0.010)	(0.97)		(0.016)	(0.97)		(0.014)	(0.95)		(0.014)	(0.96)
		GOR	-0.006	-	-	-0.003	-	-	0.040	-	-	0.011	-	-	0.009	-	-	0.034	-	-
				(0.011)	(0.87)		(0.029)	(0.93)		(0.020)	(0.47)		(0.010)	(0.73)		(0.010)	(0.81)		(0.030)	(0.84)
	t_3	MPL	0.001	0.003	0.88	0.001	0.004	0.88	0.001	0.004	0.88	0.001	0.007	0.83	0.001	0.007	0.80	0.001	0.007	0.84
				(0.004)	(0.96)		(0.005)	(0.94)		(0.005)	(0.95)		(0.007)	(0.94)		(0.006)	(0.95)		(0.006)	(0.94)
		GOR	-0.003	-	-	-0.004	-	-	0.016	-	-	0.002	-	-	0.001	-	-	0.013	-	-
				(0.005)	(0.90)		(0.016)	(0.92)		(0.012)	(0.77)		(0.004)	(0.93)		(0.004)	(0.92)		(0.022)	(0.94)

SE = standard error, CP = coverage probability

Table 5: Study 1 (partly-interval censoring): Cox and logistic regression parameters and baseline survival estimation for $n = 50$ for Scenario 1 (Weibull baseline).

			$1-\pi(\mathbf{z})=0.2$									$1-\pi(\mathbf{z})=0.6$								
			$\pi^E=0.65$			$\pi^E=0.38$			$\pi^E=0.0$			$\pi^E=0.65$			$\pi^E=0.38$			$\pi^E=0.0$		
			Bias	SE	CP	Bias	SE	CP	Bias	SE	CP	Bias	SE	CP	Bias	SE	CP	Bias	SE	CP
Regression	β_0	MPL	0.223	0.516	0.96	0.189	0.481	0.95	0.197	0.482	0.96	0.020	0.352	0.95	-0.019	0.352	0.95	0.012	0.375	0.95
			(0.149)	(0.693)		(0.126)	(0.631)		(0.131)	(0.628)		(0.040)	(0.351)		(0.037)	(0.367)		(0.025)	(0.362)	
		GOR	-0.405	0.508	0.80	-0.336	0.530	0.83	-0.314	0.611	0.81	-0.541	0.497	0.86	-0.513	0.497	0.88	-0.557	0.395	0.62
			(0.270)	(0.507)		(0.224)	(0.616)		(0.201)	(0.879)		(1.083)	(0.538)		(1.027)	(0.535)		(1.114)	(0.587)	
	β_1	MPL	0.056	0.972	0.96	0.037	0.900	0.96	0.011	0.899	0.96	0.104	0.699	0.95	0.157	0.711	0.96	0.112	0.728	0.93
			(0.056)	(0.967)		(0.037)	(1.159)		(0.011)	(0.899)		(0.104)	(0.768)		(0.157)	(0.796)		(0.112)	(0.772)	
		GOR	0.088	0.865	0.97	0.016	0.864	0.99	-0.004	1.030	0.96	0.150	0.674	0.95	0.082	0.670	0.94	0.120	0.537	0.69
			(0.088)	(0.866)		(0.016)	(0.932)		(0.004)	(0.541)		(0.150)	(0.705)		(0.082)	(0.722)		(0.120)	(0.856)	
	β_2	MPL	-0.163	0.455	0.92	-0.126	0.442	0.93	-0.117	0.447	0.93	-0.082	0.374	0.96	-0.125	0.378	0.95	-0.067	0.382	0.96
			(0.327)	(0.736)		(0.253)	(0.564)		(0.233)	(0.606)		(0.165)	(0.426)		(0.250)	(0.457)		(0.134)	(0.407)	
		GOR	-0.066	0.422	0.97	-0.120	0.444	0.96	-0.112	0.462	0.97	-0.080	0.377	0.97	-0.079	0.364	0.95	-0.075	0.288	0.72
			(0.132)	(0.433)		(0.240)	(0.541)		(0.223)	(0.594)		(0.161)	(0.617)		(0.158)	(0.410)		(0.151)	(0.444)	
	γ_1	MPL	0.006	0.322	0.89	-0.022	0.328	0.92	-0.047	0.318	0.94	0.086	0.553	0.91	0.030	0.529	0.89	-0.015	0.745	0.85
			(0.011)	(0.376)		(0.044)	(0.355)		(0.093)	(0.343)		(0.172)	(0.658)		(0.060)	(0.660)		(0.029)	(0.655)	
		GOR	0.044	0.374	0.96	0.033	0.382	0.96	-0.009	0.420	0.94	-0.108	0.587	0.94	-0.107	0.613	0.94	-0.481	0.519	0.73
			(0.087)	(0.351)		(0.066)	(0.385)		(0.017)	(0.402)		(0.216)	(0.565)		(0.213)	(0.620)		(0.962)	(0.141)	
	γ_2	MPL	0.011	0.192	0.89	-0.051	0.191	0.87	-0.036	0.181	0.89	0.112	0.349	0.85	0.094	0.322	0.85	0.042	0.489	0.84
			(0.011)	(0.242)		(0.051)	(0.221)		(0.035)	(0.225)		(0.112)	(0.414)		(0.094)	(0.427)		(0.042)	(0.359)	
		GOR	0.071	0.246	0.92	0.057	0.255	0.96	0.091	0.286	0.89	-0.097	0.383	0.86	-0.156	0.392	0.79	-0.969	0.310	0.15
			(0.071)	(0.252)		(0.057)	(0.250)		(0.091)	(0.325)		(0.097)	(0.528)		(0.156)	(0.553)		(0.969)	(0.206)	
Survival	t_1	MPL	-0.002	0.040	0.86	-0.011	0.042	0.86	-0.018	0.038	0.80	0.012	0.046	0.71	0.006	0.042	0.72	0.006	0.064	0.72
				(0.044)	(0.84)		(0.034)	(0.70)		(0.033)	(0.67)		(0.096)	(0.99)		(0.047)	(0.85)		(0.074)	(0.96)
		GOR	-0.009	-	-	-0.021	-	-	0.041	-	-	0.047	-	-	0.065	-	-	0.154	-	-
				(0.092)	(0.87)		(0.095)	(0.86)		(0.131)	(0.99)		(0.449)	(0.99)		(0.794)	(1.00)		(0.417)	(0.78)
	t_2	MPL	-0.004	0.052	0.90	-0.006	0.053	0.90	-0.007	0.051	0.87	0.001	0.087	0.87	-0.014	0.090	0.87	-0.012	0.128	0.87
				(0.078)	(0.93)		(0.069)	(0.93)		(0.069)	(0.92)		(0.201)	(1.00)		(0.097)	(0.87)		(0.143)	(0.93)
		GOR	-0.047	-	-	-0.080	-	-	0.090	-	-	-0.001	-	-	0.025	-	-	0.225	-	-
				(0.191)	(0.86)		(0.206)	(0.85)		(0.218)	(0.99)		(0.451)	(0.99)		(0.787)	(1.00)		(0.374)	(0.92)
	t_3	MPL	0.002	0.015	0.80	0.002	0.016	0.86	0.004	0.016	0.85	0.007	0.044	0.75	-0.002	0.050	0.80	-0.003	0.064	0.81
				(0.020)	(0.79)		(0.023)	(0.82)		(0.023)	(0.85)		(0.052	(0.73)		(0.056)	(0.78)		(0.082)	(0.86)
		GOR	-0.018	-	-	-0.034	-	-	0.033	-	-	-0.022	-	-	0.002	-	-	0.488	-	-
				(0.071)	(0.89)		(0.098)	(0.88)		(0.112)	(0.97)		(0.392)	(0.99)		(0.749)	(1.00)		(0.348)	(0.57)

SE = standard error, CP = coverage probability

Table 6: Study 1 (partly-interval censoring): Cox and logistic regression parameters and baseline survival estimation for $n = 50$ for Scenario 2 (exponential baseline).

			$1-\pi(\mathbf{z}) = 0.2$									$1-\pi(\mathbf{z}) = 0.6$								
			$\pi^E = 0.65$			$\pi^E = 0.38$			$\pi^E = 0.0$			$\pi^E = 0.65$			$\pi^E = 0.38$			$\pi^E = 0.0$		
			Bias	SE	CP	Bias	SE	CP	Bias	SE	CP	Bias	SE	CP	Bias	SE	CP	Bias	SE	CP
Regression	β_0	MPL	0.268	0.736	0.96	0.291	0.804	0.95	0.276	0.767	0.94	0.123	0.686	0.97	0.078	0.621	0.95	0.079	0.609	0.95
			(0.179)	(0.978)		(0.194)	(1.140)		(0.184)	(1.330)		(0.247)	(0.650)		(0.156)	(0.519)		(0.157)	(0.574)	
		GOR	-0.394	0.609	0.75	-0.386	0.579	0.82	-0.523	0.584	0.67	-1.171	5.380	0.91	-0.523	0.536	0.86	-0.697	0.419	0.61
			(0.263)	(0.757)		(0.258)	(0.626)		(0.348)	(0.828)		(2.342)	(10.942)		(1.046)	(0.624)		(1.393)	(0.632)	
	β_1	MPL	0.018	1.130	0.98	0.053	1.252	0.97	0.165	0.899	0.96	0.218	1.088	0.97	0.157	0.915	0.95	0.184	0.959	0.95
			(0.018)	(1.128)		(0.052)	(1.140)		(0.165)	(1.512)		(0.218)	(1.104)		(0.157)	(0.869)		(0.184)	(1.056)	
		GOR	0.476	9.090	0.97	0.408	6.464	0.97	-0.001	1.755	0.89	0.726	4.370	0.98	0.272	5.138	0.94	0.089	0.564	0.74
			(0.476)	(2.202)		(0.408)	(1.902)		(0.001)	(1.100)		(0.726)	(7.944)		(0.272)	(1.453)		(0.089)	(0.818)	
	β_2	MPL	-0.194	0.591	0.92	-0.211	0.608	0.93	-0.216	0.588	0.91	-0.154	0.654	0.92	-0.132	0.491	0.95	-0.145	0.530	0.93
			(0.388)	(0.813)		(0.421)	(1.065)		(0.433)	(1.317)		(0.307)	(0.869)		(0.264)	(0.619)		(0.291)	(0.679)	
		GOR	-0.115	0.504	0.95	-0.047	0.492	0.97	-0.050	0.477	0.88	-0.650	3.562	0.96	-0.146	0.403	0.95	-0.055	0.304	0.72
			(0.230)	(0.675)		(0.094)	(0.558)		(0.100)	(0.655)		(1.299)	(7.514)		(0.293)	(0.496)		(0.110)	(0.477)	
	γ_1	MPL	0.088	0.454	0.92	0.063	0.474	0.91	0.051	0.458	0.93	0.168	0.957	0.88	0.267	1.278	0.86	0.204	1.156	0.85
			(0.176)	(0.493)		(0.127)	(0.518)		(0.100)	(0.485)		(0.336)	(1.113)		(0.535)	(1.164)		(0.407)	(1.080)	
		GOR	0.075	0.395	0.93	0.080	0.407	0.96	-0.081	0.401	0.89	-0.120	0.639	0.91	-0.005	0.715	0.93	-0.428	0.547	0.73
			(0.150)	(0.422)		(0.161)	(0.422)		(0.162)	(0.485)		(0.239)	(0.630)		(0.010)	(0.708)		(0.857)	(0.316)	
	γ_2	MPL	0.196	0.288	0.91	0.180	0.298	0.91	0.175	0.292	0.91	0.508	0.809	0.85	0.432	0.898	0.89	0.303	0.847	0.87
			(0.196)	(0.335)		(0.180)	(0.338)		(0.175)	(0.320)		(0.508)	(0.776)		(0.432)	(0.720)		(0.303)	(0.672)	
		GOR	0.097	0.272	0.94	0.100	0.277	0.93	-0.131	0.264	0.74	-0.077	0.442	0.79	-0.148	0.464	0.83	-0.879	0.376	0.29
			(0.097)	(0.283)		(0.100)	(0.283)		(0.131)	(0.554)		(0.077)	(0.696)		(0.148)	(0.632)		(0.879)	(0.408)	
Survival	t_1	MPL	-0.001	0.086	0.91	0.001	0.089	0.91	-0.005	0.088	0.93	0.032	0.164	0.83	0.021	0.169	0.84	0.005	0.175	0.83
				(0.093)	(0.96)		(0.094)	(0.93)		(0.090)	(0.97)		(0.172)	(0.97)		(0.173)	(0.97)		(0.174)	(0.97)
		GOR	0.025	-	-	0.019	-	-	-0.008	-	-	-0.017	-	-	-0.034	-	-	-0.032	-	-
				(0.177)	(0.98)		(0.146)	(0.94)		(0.334)	(0.94)		(0.218)	(0.96)		(0.246)	(0.95)		(0.137)	(0.94)
	t_2	MPL	-0.014	0.057	0.79	-0.011	0.062	0.79	-0.013	0.059	0.80	0.017	0.138	0.71	0.004	0.189	0.69	-0.003	0.179	0.68
				(0.066)	(0.95)		(0.070)	(0.96)		(0.065)	(0.96)		(0.149)	(0.94)		(0.138)	(0.94)		(0.146)	(0.96)
		GOR	0.040	-	-	0.037	-	-	-0.011	-	-	-0.007	-	-	-0.023	-	-	0.013	-	-
				(0.114)	(0.98)		(0.082)	(0.94)		(0.272)	(0.94)		(0.155)	(0.91)		(0.193)	(0.95)		(0.121)	(0.94)
	t_3	MPL	-0.005	0.035	0.63	-0.002	0.039	0.63	-0.005	0.036	0.65	0.025	0.118	0.59	0.015	0.187	0.57	0.016	0.190	0.57
				(0.044)	(0.96)		(0.049)	(0.95)		(0.041)	(0.95)		(0.128)	(0.94)		(0.112)	(0.92)		(0.115)	(0.92)
		GOR	0.017	-	-	0.014	-	-	-0.025	-	-	-0.025	-	-	-0.036	-	-	0.019	-	-
				(0.085)	(0.98)		(0.042)	(0.95)		(0.240)	(0.94)		(0.117)	(0.93)		(0.159)	(0.94)		(0.103)	(0.94)

SE = standard error, CP = coverage probability

Table 7: Study 1 (partly-interval censoring): Cox and logistic regression parameters and baseline survival estimation for $n = 50$ for Scenario 3 (log-logistic baseline).

			$1-\pi(\mathbf{z}) = 0.2$									$1-\pi(\mathbf{z}) = 0.6$								
			$\pi^E = 0.65$			$\pi^E = 0.38$			$\pi^E = 0.0$			$\pi^E = 0.65$			$\pi^E = 0.38$			$\pi^E = 0.0$		
			Bias	SE	CP	Bias	SE	CP	Bias	SE	CP	Bias	SE	CP	Bias	SE	CP	Bias	SE	CP
Regression	β_0	MPL	0.177	0.815	0.94	0.089	0.611	0.93	0.129	0.596	0.95	0.042	0.461	0.94	0.050	0.449	0.96	-0.006	0.472	0.95
			(0.118)	(0.944)		(0.059)	(0.721)		(0.086)	(0.651)		(0.083)	(0.512)		(0.100)	(0.480)		(0.013)	(0.415)	
		GOR	-0.268	7.328	0.81	-0.443	0.587	0.75	-0.403	0.589	0.76	-0.621	0.538	0.86	-0.559	0.607	0.86	-0.725	1.139	0.67
			(0.179)	(1.310)		(0.295)	(0.667)		(0.268)	(0.770)		(1.241)	(0.579)		(1.118)	(0.611)		(1.450)	(2.067)	
	β_1	MPL	-0.004	1.106	0.95	-0.087	1.025	0.96	0.007	0.997	0.97	0.087	0.829	0.94	0.132	0.779	0.96	0.107	0.807	0.95
			(0.004)	(1.027)		(0.087)	(0.975)		(0.007)	(0.935)		(0.087)	(1.095)		(0.132)	(0.968)		(0.107)	(0.797)	
		GOR	0.181	7.131	0.96	0.234	8.071	0.97	-0.057	1.621	0.95	0.226	0.752	0.95	0.132	0.804	0.95	0.311	3.399	0.78
			(0.181)	(1.700)		(0.234)	(1.588)		(0.057)	(1.232)		(0.226)	(0.924)		(0.132)	(0.835)		(0.311)	(5.448)	
	β_2	MPL	-0.181	0.635	0.93	-0.080	0.500	0.93	-0.148	0.516	0.91	-0.126	0.447	0.95	-0.113	0.423	0.96	-0.066	0.413	0.95
			(0.363)	(0.842)		(0.160)	(0.682)		(0.296)	(0.660)		(0.253)	(0.621)		(0.226)	(0.577)		(0.132)	(0.447)	
		GOR	-0.158	0.484	0.94	-0.079	0.478	0.94	-0.143	0.503	0.92	-0.126	0.404	0.96	-0.078	0.429	0.95	-0.359	4.070	0.78
			(0.316)	(0.582)		(0.158)	(0.553)		(0.286)	(0.667)		(0.251)	(0.484)		(0.156)	(0.455)		(0.718)	(6.618)	
	γ_1	MPL	0.085	0.410	0.90	0.058	0.454	0.92	0.049	0.406	0.93	0.166	1.253	0.86	0.168	0.833	0.87	0.071	0.784	0.90
			(0.171)	(0.479)		(0.117)	(0.465)		(0.097)	(0.418)		(0.332)	(0.993)		(0.336)	(0.878)		(0.142)	(0.841)	
		GOR	0.075	0.387	0.93	0.010	0.397	0.93	-0.021	0.421	0.94	-0.170	0.648	0.93	-0.077	0.747	0.93	-0.447	0.560	0.77
			(0.150)	(0.435)		(0.020)	(0.477)		(0.042)	(0.430)		(0.340)	(0.697)		(0.154)	(0.779)		(0.894)	(0.266)	
	γ_2	MPL	0.110	0.282	0.90	0.106	0.284	0.92	0.077	0.270	0.91	0.351	0.987	0.85	0.256	0.571	0.83	0.235	0.691	0.84
			(0.110)	(0.317)		(0.106)	(0.299)		(0.077)	(0.289)		(0.351)	(0.671)		(0.256)	(0.576)		(0.235)	(0.538)	
		GOR	0.107	0.261	0.91	0.085	0.264	0.91	-0.049	0.274	0.81	-0.186	0.464	0.78	-0.091	0.504	0.85	-0.892	0.388	0.26
			(0.107)	(0.308)		(0.085)	(0.295)		(0.049)	(0.446)		(0.186)	(0.657)		(0.091)	(0.729)		(0.892)	(0.385)	
Survival	t_1	MPL	0.011	0.079	0.92	0.017	0.084	0.95	0.016	0.077	0.92	0.033	0.151	0.84	0.019	0.163	0.85	0.010	0.171	0.86
				(0.120)	(1.00)		(0.114)	(0.99)		(0.116)	(0.99)		(0.203)	(0.98)		(0.193)	(0.99)		(0.197)	(1.00)
		GOR	0.051	-	-	0.086	-	-	0.089	-	-	0.021	-	-	0.028	-	-	-0.012	-	-
				(0.140)	(0.88)		(0.156)	(0.90)		(0.150)	(0.95)		(0.233)	(0.88)		(0.235)	(0.90)		(0.175)	(0.98)
	t_2	MPL	-0.011	0.039	0.69	-0.010	0.052	0.74	-0.008	0.040	0.70	0.010	0.134	0.64	0.001	0.127	0.63	-0.006	0.123	0.64
				(0.058)	(0.98)		(0.065)	(0.96)		(0.059)	(0.96)		(0.144)	(0.97)		(0.142)	(0.96)		(0.141)	(0.97)
		GOR	0.006	-	-	0.022	-	-	0.054	-	-	-0.044	-	-	-0.037	-	-	0.036	-	-
				(0.070)	(0.91)		(0.076)	(0.92)		(0.104)	(0.93)		(0.154)	(0.90)		(0.178)	(0.89)		(0.164)	(0.96)
	t_3	MPL	-0.006	0.016	0.44	-0.007	0.022	0.51	0.006	0.015	0.48	0.010	0.108	0.44	0.004	0.070	0.43	-0.002	0.067	0.43
				(0.025)	(0.94)		(0.030)	(0.96)		(0.026)	(0.95)		(0.098)	(0.95)		(0.094)	(0.93)		(0.082)	(0.94)
		GOR	-0.003	-	-	0.004	-	-	0.025	-	-	-0.044	-	-	-0.045	-	-	0.032	-	-
				(0.040)	(0.95)		(0.035)	(0.94)		(0.063)	(0.93)		(0.109)	(0.90)		(0.132)	(0.91)		(0.150)	(0.94)

SE = standard error, CP = coverage probability

Table 8: Study 1 (partly-interval censoring): Cox and logistic regression parameters and baseline survival estimation for $n = 200$ and $n = 1000$ for Weibull baseline and 5% cured.

			$1 - \pi(\mathbf{z}) = 0.05$								
			$\pi^E = 0.65$			$\pi^E = 0.38$			$\pi^E = 0.0$		
			$n = 200$								
			Bias	SE	CP	Bias	SE	CP	Bias	SE	CP
Regression	β_0	MPL	0.148	0.409	0.96	0.124	0.405	0.97	0.215	0.455	0.96
			(0.049)	(0.487)		(0.041)	(0.391)		(0.072)	(0.542)	
	β_1	MPL	0.108	0.751	0.97	0.066	0.745	0.98	0.071	0.828	0.96
			(0.108)	(0.869)		(0.066)	(0.772)		(0.071)	(0.992)	
	β_2	MPL	-0.059	0.316	0.92	-0.023	0.319	0.94	-0.051	0.331	0.92
			(0.119)	(0.350)		(0.045)	(0.338)		(0.102)	(0.373)	
	γ_1	MPL	-0.004	0.148	0.95	-0.006	0.151	0.93	-0.012	0.152	0.94
			(0.008)	(0.150)		(0.011)	(0.164)		(0.024)	(0.163)	
	γ_2	MPL	-0.001	0.090	0.90	-0.010	0.092	0.92	-0.008	0.093	0.91
			(0.001)	(0.102)		(0.010)	(0.100)		(0.008)	(0.105)	
Survival	t_1	MPL	-0.004	0.029	0.93	-0.003	0.028	0.93	-0.004	0.029	0.93
				(0.043)	(0.99)		(0.050)	(0.99)		(0.059)	(0.94)
	t_2	MPL	-0.001	0.013	0.88	0.001	0.015	0.90	0.001	0.015	0.88
				(0.022)	(0.94)		(0.031)	(0.98)		(0.120)	(0.92)
	t_3	MPL	0.001	0.001	0.84	0.001	0.001	0.86	0.001	0.001	0.85
				(0.001)	(0.84)		(0.001)	(0.88)		(0.070)	(0.85)
			$n = 1000$								
			Bias	SE	CP	Bias	SE	CP	Bias	SE	CP
Regression	β_0	MPL	0.036	0.168	0.95	0.048	0.170	0.94	0.014	0.167	0.95
			(0.012)	(0.177)		(0.016)	(0.182)		(0.005)	(0.168)	
	β_1	MPL	0.025	0.312	0.96	0.053	0.315	0.93	0.021	0.310	0.93
			(0.025)	(0.306)		(0.053)	(0.335)		(0.021)	(0.343)	
	β_2	MPL	-0.010	0.141	0.95	0.001	0.143	0.93	0.004	0.141	0.95
			(0.020)	(0.142)		(0.001)	(0.147)		(0.007)	(0.135)	
	γ_1	MPL	-0.005	0.067	0.94	-0.005	0.068	0.95	0.012	0.069	0.95
			(0.010)	(0.067)		(0.010)	(0.067)		(0.024)	(0.070)	
	γ_2	MPL	-0.006	0.042	0.93	-0.006	0.043	0.92	-0.005	0.044	0.94
			(0.006)	(0.045)		(0.006)	(0.048)		(0.006)	(0.043)	
Survival	t_1	MPL	-0.001	0.020	0.95	-0.002	0.015	0.92	-0.001	0.015	0.97
				(0.028)	(0.99)		(0.027)	(1.00)		(0.030)	(1.00)
	t_2	MPL	0.001	0.002	0.93	0.001	0.002	0.93	0.001	0.003	0.92
				(0.003)	(0.99)		(0.004)	(0.98)		(0.005)	(0.98)
	t_3	MPL	0.001	0.001	0.91	0.001	0.001	0.89	0.001	0.001	0.92
				(0.001)	(0.92)		(0.001)	(0.89)		(0.001)	(0.90)

References

[1] Ma J, Couturier D-L, Heritier S, Marschner I. Proportional hazards model with partly interval censoring and its penalized likelihood estimation. *arXiv preprint arXiv:1904.06789.* 2019.
